# A Systematic Review of *Campylobacter jejuni* Vaccine Candidates for Chickens

**DOI:** 10.3390/microorganisms9020397

**Published:** 2021-02-15

**Authors:** Pongthorn Pumtang-on, Timothy J. Mahony, Rodney A. Hill, Thiru Vanniasinkam

**Affiliations:** 1 School of Biomedical Sciences, Charles Sturt University, Wagga Wagga, NSW 2650, Australia; pongthornp7@gmail.com (P.P.-o.); rhill@csu.edu.au (R.A.H.); 2 Centre for Animal Science, Queensland Alliance for Agriculture and Food Innovation, The University of Queensland, Brisbane, QLD 4072, Australia; t.mahony@uq.edu.au

**Keywords:** *Campylobacter jejuni*, vaccination, poultry, chickens, systematic review

## Abstract

*Campylobacter jejuni* infection linked to the consumption of contaminated poultry products is one of the leading causes of human enteric illness worldwide. Vaccination of chickens is one of the potential strategies that could be used to control *C. jejuni* colonization. To date, various *C. jejuni* vaccines using potential antigens have been evaluated, but a challenge in identifying the most effective formulation is the wide variability in vaccine efficacies reported. A systematic review was undertaken to compare *C. jejuni* vaccine studies. Based upon specific selection criteria eligible papers were identified and included in the analysis. Vaccine efficacy reported from different *C. jejuni* antigens, vaccine types, and vaccination regimens reported in these papers were reviewed. Our analysis shows that total outer membrane proteins and cysteine ABC transporter substrate-binding protein were among the most efficacious vaccine antigen candidates reported. This review also highlights the importance of the need for increased consistency in the way *C. jejuni* vaccine studies in poultry are designed and reported in order to be able to undertake a robust comparison of *C. jejuni* vaccine candidates.

## 1. Introduction

*Campylobacter jejuni* is considered an important zoonotic pathogen causing enteric illness in humans globally [1,2,3]. Outbreaks are commonly linked to the consumption of contaminated poultry products [4,5,6]. Poultry is considered a reservoir host of *C. jejuni* because this pathogen commensally colonizes the intestines where it can be present in large bacterial loads [7]. Based on quantitative risk assessment and regression models in previous studies, a low *C. jejuni* prevalence (a percentage/proportion of colonized chickens in a flock) between chicken flocks or a 1 to 2 log10 reduction of *C. jejuni* loads in broiler intestines could lead to a decrease in public health risk [8,9,10]. Thus, both the reduction in *C. jejuni* concentration and prevention of campylobacter colonization of chickens on farms are the most effective approaches to reduce the risk of campylobacter contamination of chicken meat [9]. To date, researchers have endeavored to develop and evaluate several interventions in primary broiler production including biosecurity monitoring [11], use of feed additives [12,13,14], drinking water sanitation [15], use of bacteriophage [16], probiotics [17,18], and bacteriocins [19]. Although some of these interventions have led to significant reductions in *C. jejuni* loads in the intestines of chickens, none of them have eliminated or prevented *C. jejuni* colonization of poultry.

Vaccination has been considered a potentially effective intervention for controlling *C. jejuni* colonization of chickens. In recent decades, *C. jejuni* has been extensively studied, with various prototype vaccines containing potential *C. jejuni* antigens being evaluated [20,21,22,23,24,25,26,27,28,29,30]. While these studies have typically reported that the prototype vaccines have elicited strong immunogen specific immune responses, they have concurrently reported variable outcomes with respect to vaccine efficacy. Typically, these vaccine efficacies would be insufficient in reducing *C. jejuni* concentration in gut samples and/or preventing colonization. As a consequence, vaccines to prevent *C. jejuni* colonization of chickens are yet to become commercially available.

This review aimed to summarize published studies on vaccines to prevent *C. jejuni* colonization in chickens using a systematic review approach and identify vaccine antigens most suitable for further development.

## 2. Materials and Methods

A systemic review was carried out according to the guidelines of the Preferred Reporting Items for Systematic Reviews and Meta-Analyzes (PRISMA) [31]. The following research questions were considered in this review.

Key research questions:

1. What antigens have been identified for use in potential vaccine candidates to prevent *C. jejuni* colonization in chickens?

2. What are the most efficacious *C. jejuni* vaccine candidates in chickens identified?

### 2.1. Search Strategy (Literature Search Strategies/Identifying Data Source)

#### 2.1.1. Databases Searched

Three electronic databases, PubMed Central, Scopus, and Elsevier ScienceDirect, were searched to identify relevant studies for this review.

#### 2.1.2. Keywords Used in the Search

The search was performed on January 4, 2021, using the following terms: “*Campylobacter*” AND “Vaccine” AND “Chicken” (Table 1). All research articles were restricted to English language only, all fields, and were previously published until 2020 (31 December).

### 2.2. Selection Criteria

Eligibility of studies for inclusion in this review was determined using a two-step process (primary and secondary inclusion/exclusion criteria) (Table 2).

Vaccine trials using layers were excluded in this review due to reported differences in *C. jejuni* colonization and immune responses between layer and broiler chicken breeds [32,33] and an expectation of reducing the public health risk [9,34]. If more than one sample type (i.e., ceca and cloaca) were evaluated in a single study, the result for the cecal sample was selected for the purposes of this study [30]. In applying this, we have not differentiated trials using conventional (bacterial culture) and/or molecular techniques (i.e., qPCR) to determine the colonization status of chickens at the end of vaccination studies [35,36]. Previous studies reported that both bacterial culture and qPCR methods had a high correlation (>99%) for enumerating *C. jejuni* in intestinal samples [22] and no significant difference in the detection of *Campylobacter* in chicken faecal samples [37]. Studies where the required details for the vaccine efficacy data were not provided in a usable format, the author (P.P.) contacted the study corresponding author of the original articles requesting the missing data via email two times. None of the corresponding authors responded to these requests.

#### Defining Vaccine Efficacy for Selecting Eligible Studies for This Review

The effectiveness of various *C. jejuni* controlling interventions at broiler farms is commonly evaluated using reduction levels in the prevalence of colonized chickens in a flock and/or reductions of *C. jejuni* loads in the broiler intestine at slaughterhouse [9]. Previous studies, EFSA [9] and Rosenquist et al. [34] have reported that a decrease in the prevalence of *C. jejuni* between and within broiler flocks could reduce bacterial loads in carcasses at slaughter and consequently reduce the incidence of human campylobacteriosis. Moreover, Nauta et al. [10] reported that a 1–2 log reduction in *C. jejuni* loads in gut contents had an impact on the human health risk of campylobacteriosis with a relative risk reduction by at least 44% based on regression and risk assessment models. Therefore, articles reporting vaccine efficacy based upon prevalence or proportion of “diseased” (i.e., colonized) chickens in a flock or group [38] and/or the reduction levels of *C. jejuni* colonization in vaccinated and unvaccinated chicken groups after *C. jejuni* challenge, were included in this review.

### 2.3. Data Extraction

All research articles identified from the three databases were entered in Microsoft Excel datasheets and duplicate studies were removed by one author (P.P.). One author (P.P.) initially inspected the titles and abstracts from the individual articles to select articles for inclusion in the review. If those titles and abstracts fitted the selection criteria, the full text of each potential article was further examined for the final determinations of eligible studies. At this stage, the full text was reviewed to classify the eligible studies and trials based on the vaccine-controlled efficacy trials described and to extract the relevant information. Subsequently, another independent reviewer (T.V.) validated the data and results. For any disagreements, all conflicts were resolved by consensus, and a third author (T.J.M. or R.A.H.) was asked to confirm whether the articles should be included or excluded. The final lists of the eligible article were imported to the EndnoteX9 program for storage, and consolidation (P.P.).

The extracted information of the individual eligible studies included article identification (authors and publication year), the title of each article, study type, information of animal models (poultry species), vaccine types, vaccine regimen (dosages of vaccine and adjuvants, ages of chickens, antigen candidates, frequency of vaccination, and bacterial challenge strains), samples collected, isolation test and outcome measurements of vaccine efficacy between the vaccinated and unvaccinated groups at the end of study. The concentration of *C. jejuni loads* in cecal contents and/or reduction levels of *C. jejuni* colonization reported in text and tabulation and/or estimated from figures provided in the original papers were included in this review. For multiple trials reported in each paper, each trial was considered as a separate trial unless the trials using the same vaccine protocols and evaluation methods. If only one control group was used to compare with more than one type of vaccine in the same experiment, this control group was used for each comparison. If two control groups were used in the DNA vaccine study, one control group with the parent plasmids (no insertion of an antigen of interest) was used as the control group [39]. The extracted information was summarized in Microsoft Excel datasheets.

### 2.4. Data Analysis

The extracted data were analysed with the aim of conducting a systematic review and/or meta-analysis. Based on the data extracted using a definition of prevalence detected in vaccinated broilers, six eligible articles (18 trials) reporting different *C. jejuni* antigens, vaccine types, and vaccine protocols were identified. Consequently, it was possible to undertake a systematic review, but the data were insufficient to conduct a meta-analysis. The outcomes of individual vaccine trials of the eligible studies were extracted, analysed, and reported as a percentage (proportion) of colonized broilers and relative risk (RR) with 95% confidence interval (CI). Trials with RR < 1.00 were further analysed with respect to vaccine efficacy as it indicates that the exposed (vaccinated) group could reduce a ratio of the risk or possibility of disease (*C. jejuni*) occurrence, compared to the unexposed (non-vaccinated) group [40]. While trials with RR is ≥ 1.00 were reported as having no effect in this review. The efficacy of vaccine was calculated as (1 − RR) and reported as a percentage [39,41,42]. The R software program (Version 1.3.1056, the R Foundation, Vienna, Austria) was used for calculating these estimates [43].

Trials demonstrating a log10 reduction of *C. jejuni* loads between vaccinated and non-vaccinated broilers and reporting this as a geometric or arithmetic mean or median of log10 (CFU/gram) loads of each treatment group were included. Extracted data from 62 trials reported in 16 papers were included for this review.

## 3. Results

### 3.1. Search Results

**Figure 1:**
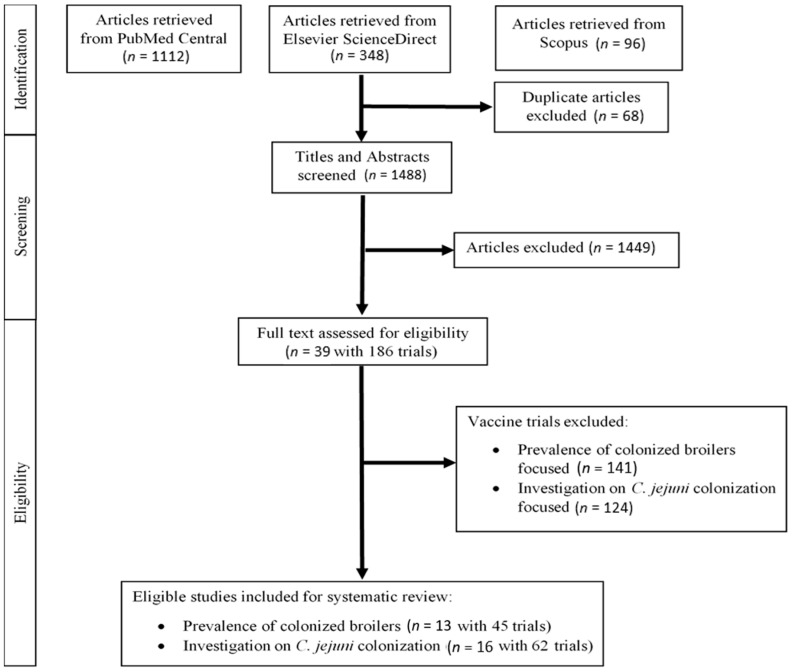
Flow diagram of the selection process to identify articles to be included in the systematic review.

A total of 1556 articles were retrieved from the three electronic databases (PubMed Central, Scopus, and Elsevier ScienceDirect). Of these, 1488 articles (95.6%) were assessed using the text of the title and abstract after the removal of duplicates. The selection process used in the current study is shown in Figure 1.

#### 3.1.1. Screening Process

Following the screening of titles and abstracts, 1449 articles were excluded (Figure 1). Of the excluded articles, 557 were review articles: 122 (*Campylobacter* biology, pathogenesis, genes, control and prevention, and vaccines), 95 (human infectious diseases and communicable disease), 78 (antimicrobials and probiotics), 67 (micro-organism: microbial, virus, bacteriophages protozoa and parasites), 49 (bacteria other than *Campylobacter*), 47 (immunity and antigens), 35 (zoonosis, plant-based, and dietary), 21 (foodborne, waterborne, and food safety), 16 (genetic controls, guidelines of prevention and diagnosis, internal organs, and toxins), 13 (methods and biotechnologies), seven (birds, turkey, ferret, ruminant, and rabbit), six (cancers, chicken gastrointestinal tract, poultry management, taxonomy, and wastewater), and one (no author name provided). A total of 877 primary research studies did not meet the selection criteria: 538 (non-*Campylobacter* studies), 237 (*Campylobacter* studies but not vaccines), 85 (non-*Campylobacter* vaccine studies), and 17 (*Campylobacter* vaccines conducted in non-poultry animals, immunogenicity experiments, and unable to obtain full-text). In addition, 12 and three excluded articles were proceeding abstracts and non-English language, respectively.

#### 3.1.2. Eligibility

The remaining 39 articles fulfilled the initial selection criteria for further assessing the full text for eligibility and were published within the search period. From the 37 articles, 186 trials were identified, and these trials involved the evaluation of *C. jejuni* vaccines conducted in chickens (layers and broilers). Of the 186 trials, 66 trials conducted in layer chickens were excluded. Thus, 120 trials conducted in broilers were included for further review using two different focuses of vaccine efficacy (the prevalence of colonized broilers and significant log10 reduction levels) reported in text/tabulations of the original papers. 

Based on the full-text evaluation using the investigation on the *C. jejuni* loads in cecal contents, 58 of 120 vaccine trials in broilers were excluded as they were a seeder colonization challenge model (*n* = 22), immunogenicity studies (*n* = 18), vaccine efficacy evaluated from ileum and cloaca (*n* = 15), trials using co-administration of vaccine and probiotics (*n* = 2), vaccine efficacy reported as prevalence (*n* = 1). Consequently, 62 vaccine trials fulfilled the selection criteria of this review. A summary of the details of these studies is shown in Table 3.

Based on the full-text evaluation using the prevalence of colonized chickens, 75 of 120 trials (85.0%) were excluded. The majority of the excluded trials were vaccine trials using a seeder colonization challenge model (*n* = 22), followed by data of number of individual colonized broiler not reported or unable to estimate from figures (*n* = 18), immune responses reported (*n* = 18), vaccine efficacy evaluated from ileum and cloaca (*n* = 15; 4 articles) [22,28,44,45], and trials using co-administration of vaccine and probiotics (*n* = 2; 1 article) [46]. The remaining 45 trials (10.1%) from 13 articles fulfilled the selection criteria and were included in the systematic review. 

**Table 3:** Summary of *Campylobacter jejuni* vaccine trials in broilers evaluated based on isolation of *Campylobacter jejuni* from cecal contents.

Trial No.	Vaccine Formulations, Antigens, and Regimens	Bacterial Challenge Strain (Dose (log10 CFU)) and Day of Challenge	Age of Chickens at the End of Study (Days)	Levels of *Campylobacter jejuni* Colonization (Mean log10 CFU/gram ^1^ and/or ± Standard Error of Mean) Following Challenge		Reduction in Levels (Mean log10 CFU/gram) of *C. jejuni* Colonization Reported ^2^	Reference
				Vaccinated Broilers	Non-Vaccinated Broilers		
**1**	Crude cell lysate vaccine with 125 µg of total outer membrane proteins (OMP) encapsulated with poly lactide-co-glycolide nanoparticles (OMP-NP), orally with booster	*C. jejuni* 81–176 (8.0) and Day 35	42	6.3 ^3^	6.7 ^3^	Non-significant 0.4 log 10 reduction	Annamalai et al. [47]
**2**	Crude cell lysate vaccine with 125 µg of OMP, orally with booster	*C. jejuni* 81–176 (8.0) and Day 35	42	5.9 ^3^	6.7 ^3^	Non-significant 0.8 log10 reduction ^4^	Annamalai et al. [47]
**3**	Crude cell lysate vaccine with 125 µg of OMPs-NP, subcutaneously with booster	*C. jejuni* 81–176 (8.0) and Day 35	42	<1.00 (below detection limit)	6.7 ^3^	Significant 5.7 log10 reductions	Annamalai et al. [47]
**4**	Crude cell lysate vaccine with 125 µg of OMP, orally with booster	*C. jejuni* 81–176 (8.0) and Day 35	42	<1.00 (below detection limit)	6.7 ^3^	Significant 5.7 log10 reductions ^4^	Annamalai et al. [47]
**5**	Crude cell lysate vaccine with 25 µg of OMP-NP, subcutaneously with booster	*C. jejuni* 81–176 (8.0) and Day 35	42	5.5 ^3^	6.7 ^3^	Non-significant 1.2 log10 reductions	Annamalai et al. [47]
**6**	Crude cell lysate vaccine with 250 µg of OMP-NP, orally with booster	*C. jejuni* 81–176 (8.0) and Day 35	42	5.8 ^3^	6.7 ^3^	Non-significant 0.9 log10 reductions	Annamalai et al. [47]
**7**	Crude cell lysate vaccine with 25 µg of OMP, orally with booster	*C. jejuni* 81–176 (8.0) and Day 35	42	5.1 ^3^	6.7 ^3^	Non-significant 1.6 log10 reductions ^4^	Annamalai et al. [47]
**8**	Crude cell lysate vaccine with 250 µg of OMP, orally with booster	*C. jejuni* 81–176 (8.0) and Day 35	42	5.7 ^3^	6.7 ^3^	Non-significant 1.0 log10 reduction ^4^	Annamalai et al. [47]
**9**	10^9^ CFU of *L. lactis* NZ9000 strain vectored vaccine expressing *C. jejuni* surface-exposed lipoprotein A (JlpA), intragastrically with booster	*C. jejuni* BCH71 (9.0) and Day 28	35	6.43 ± 0.107 in Trial#1 8.06 ± 0.05 in Trial#2 9.078 ± 0.052 in Trial#3	7.22 ± 0.106 in Trial#1 8.53 ± 0.089 in Trial#2 9.56 ± 0.075 in Trial#3	Significant 0.79 log10 reduction in Trial#1 Significant 0.47 log10 reduction in #2 Significant 0.482 log10 reduction in #3	Gorain et al. [48]
**10**	Subunit vaccine with 50 µg of recombinant JlpA emulsified in Freund’s incomplete adjuvant, subcutaneously with booster	*C. jejuni* BCH71 (9.0) and Day 28	35	6.89 ± 0.091 in Trial#1 7.90 ± 0.05 in Trial#2 9.15 ± 0.080 in Trial#3	7.00 ± 0.107 in Trial#1 8.59 ± 0.069 in Trial#2 9.64 ± 0.037 in Trial#3	Non-significant 0.11 log10 in Trial#1 Significant 0.69 log10 in Trial#2 Significant 0.49 log10 in Trial#3	Gorain et al. [48]
**11**	25 µg of Capsular polysaccharide conjugated with diphtheria toxoid of *Corynebacterium diphtheriae* vaccine (CPSconj) mixed with 10 µg of CpG ODN 2007, subcutaneously with booster	*C. jejuni* 81–176 (7.3) and Day 28	38	7.55 ± 0.15	8.11 ± 0.15	Significant 0.56 log10 reduction ^5^	Hodgins et al. [49]
**12**	25 µg of CPSconj mixed with 100 µL of Addavax, subcutaneously with booster	*C. jejuni* 81–176 (7.3) and Day 28	38	7.47 ± 0.14	8.11 ± 0.15	Significant 0.64 log10 redcution ^5^	Hodgins et al. [49]
**13**	25 µg of CPSconj, subcutaneously with booster	*C. jejuni* 81–176 (7.3) and Day 28	38	7.38 ± 0.15	8.11 ± 0.15	Significant 0.73 log10 reduction	Hodgins et al. [49]
**14**	DNA vaccine (prime) with 300 µg of purified DNA of *Campylobacter* hemolysin activation/secretion protein (YP_001000437.1) cloned into pcDNA3 plasmids mixed with 50 µg of CpG ODN2007 and subunit vaccine (boost) with 100 µg of recombinant YP_001000437.1 protein emulsified with MONTANIDE™ ISA70 VG, intramuscularly with booster	*C. jejuni* C97Anses640 (5.0) and Day 19	42	4.41 ± 2.15	8.02 ± 1.19	Significant 3.61 log10 reductions upon heterologous challenge	Meunier et al. [50]
**15**	DNA vaccine (prime) with 300 µg of purified DNA of YP_001000437.1 cloned into pcDNA3 plasmids mixed with 50 µg of CpG ODN2007 and subunit vaccine (boost) with 100 µg of recombinant YP_001000437.1 protein emulsified with MONTANIDE™ ISA70 VG, intramuscularly with booster	*C. jejuni* C97Anses640 (5.0) and Day 19	42	3.53 ± 1.86 (GenEq/g)	5.45 ± 2.61 (GenEq/g)	Non-significant 1.92 log10 GenEq/g reductions upon heterologous challenge	Meunier et al. [50]
**16**	DNA vaccine (prime) with 300 µg of purified DNA of flagellin protein family (FlgL) cloned into pcDNA3 plasmids mixed with 50 µg of CpG ODN2007 and subunit vaccine (boost) with 100 µg of recombinant FlgL emulsified with MONTANIDE™ ISA70 VG, intramuscularly with booster	*C. jejuni* C97Anses640 (5.0) and Day 19	42	5.99 ± 1.48	8.02 ± 1.19	Significant 2.03 log10 reductions upon heterologous challenge	Meunier et al. [50]
**17**	DNA vaccine (prime) with 300 µg of purified DNA of FlgL cloned into pcDNA3 plasmids mixed with 50 µg of CpG ODN2007 and subunit vaccine (boost) with 100 µg of recombinant FlgL emulsified with MONTANIDE™ ISA70 VG, intramuscularly with booster	*C. jejuni* C97Anses640 (5.0) and Day 19	42	4.39 ± 2.37 (GenEq/g)	5.45 ± 2.61 (GenEq/g)	Non-significant 1.06 log10 GenEq/g reductions upon heterologous challenge	Meunier et al. [50]
**18**	DNA vaccine (prime) with 300 µg of purified DNA of hypothetical protein (YP99838.1) cloned into pcDNA3 plasmids mixed with 50 µg of CpG ODN2007 and subunit vaccine (boost) with 100 µg of recombinant YP99838.1 emulsified with MONTANIDE™ ISA70 VG, intramuscularly with booster	*C. jejuni* C97Anses640 (5.0) and Day 19	42	5.94 ± 1.48	8.02 ± 1.19	Significant 2.08 log10 reductions upon heterologous challenge	Meunier et al. [50]
**19**	DNA vaccine (prime) with 300 µg of purified DNA of YP99838.1 cloned into pcDNA3 plasmids mixed with 50 µg of CpG ODN2007 and subunit vaccine (boost) with 100 µg of YP99838.1 emulsified with MONTANIDE™ ISA70 VG, intramuscularly with booster	*C. jejuni* C97Anses640 (5.0) and Day 19	42	6.83 ± 0.91 (GenEq/g)	5.45 ± 2.61 (GenEq/g)	No reduction upon heterologous challenge	Meunier et al. [50]
**20**	DNA vaccine (prime) with 300 µg of purified DNA of hypothetical protein (YP99817.1) cloned into pcDNA3 plasmids mixed with 50 µg of CpG ODN2007 and subunit vaccine (boost) with 100 µg of recombinant YP99817.1 emulsified with MONTANIDE™ ISA70 VG, intramuscularly with booster	*C. jejuni* C97Anses640 (5.0) and Day 19	42	3.75 ± 1.49	8.02 ± 1.19	Significant 4.27 log10 reductions upon heterologous challenge	Meunier et al. [50]
**21**	DNA vaccine (prime) with 300 µg of purified DNA of YP99817.1 cloned into pcDNA3 plasmids mixed with 50 µg of CpG ODN2007 and subunit vaccine (boost) with 100 µg of recombinant YP99817.1 emulsified with MONTANIDE™ ISA70 VG, intramuscularly with booster	*C. jejuni* C97Anses640 (5.0) and Day 19	42	6.19 ± 2.16 (GenEq/g)	5.45 ± 2.61 (GenEq/g)	No reduction upon heterologous challenge	Meunier et al. [50]
**22**	DNA vaccine with 300 µg of purified DNA of YP99817.1 cloned into pcDNA3 plasmids mixed with 50 µg of CpG ODN2007, intramuscularly with booster	*C. jejuni* C97Anses640 (5.0) and Day 19	42	7.04	6.2 ^3^	No reduction upon heterologous challenge	Meunier et al. [50]
**23**	Subunit vaccine with 100 µg of recombinant YP99817.1 emulsified with MONTANIDE™ ISA70 VG, intramuscularly with booster	*C. jejuni* C97Anses640 (5.0) and Day 19	42	7.87	7.03	No reduction upon heterologous challenge	Meunier et al. [50]
**24**	DNA vaccine (prime) with 300 µg of purified DNA of flagellar hook-basal body complex protein (FlgE-1) cloned into pcDNA3 plasmids mixed with 50 µg of CpG ODN2007 and subunit vaccine (boost) with 100 µg of recombinant FlgE-1 emulsified with MONTANIDE™ ISA70 VG, intramuscularly with booster	*C. jejuni* C97Anses640 (5.0) and Day 19	42	5.8 ^3^	8.02 ± 1.19	Non-significant 2.20 log10 reductions (a wide range of individual colonized broilers was observed in the work of Meunier et al.) upon heterologous challenge	Meunier et al. [50]
**25**	DNA vaccine (prime) with 300 µg of purified DNA of flagellar hook-associated protein (FlgK) cloned into pcDNA3 plasmids mixed with 50 µg of CpG ODN2007 and subunit vaccine (boost) with 100 µg of recombinant FlgK emulsified with MONTANIDE™ ISA70 VG, intramuscularly with booster	*C. jejuni* C97Anses640 (5.0) and Day 19	42	6.3 ^3^	8.02 ± 1.19	Non-significant 1.72 log10 reductions (a wide range of individual colonized broilers was observed in the work of Meunier et al.) upon heterologous challenge	Meunier et al. [50]
**26**	DNA vaccine (prime) with 300 µg of multiple DNA proteins (a combination of purified YP_001000437.1, FlgL, FlgK, FliE-1, YP99817.1, and YP99838.1) cloned into pcDNA3 plasmids mixed with 50 µg of CpG ODN2007 and subunit vaccine (boost) with 100 µg of recombinant multiple proteins (YP_001000437.1, FlgL, FlgK, FliE-1, YP99817.1, and YP99838.1) emulsified with MONTANIDE™ ISA70 VG, intramuscularly with booster	*C. jejuni* C97Anses640 (5.0) and Day 19	42	7.9 ^3^	8.02 ± 1.19	Non-significant 0.12 log10 reduction (No decrease of *C. jejuni* colonization reported in the original paper) upon heterologous challenge	Meunier et al. [50]
**27**	DNA vaccine with 100 µg of purified DNA of flagellin A protein (FlaA) cloned into pcDNA3 plasmid mixed with 25 µg of CpG ODN2007, subcutaneously with booster	*C. jejuni* 81–176 (5.0) and Day 21	42	7.7 ^3^ (geometric mean)	7.8 ^3^ (geometric mean)	Non-significant 0.1 geometric mean log10 reduction	Meunier et al. [51]
**28**	DNA vaccine with 100 µg of purified DNA of FlaA cloned into pcDNA3 plasmid mixed with 25 µg of CpG ODN2007, intramuscularly with booster	*C. jejuni* 81–176 (5.0) and Day 21	42	5.0 ^3^ (geometric mean)	5.2 ^3^ (geometric mean)	Non-significant 0.2 median log10 reductions	Meunier et al. [51]
**29**	DNA vaccine (prime) with 150 µg of purified DNA of FlaA into pcDNA3 plasmid mixed with 25 µg of CpG ODN2007 and subunit vaccine (boost) with 100 µg of recombinant FlaA emulsified with MONTANIDE™ ISA70 VG, intramuscularly with booster	*C. jejuni* 81–176 (5.0) and Day 21	42	5.3 ^3^ (geometric mean)	5.2 ^3^ (geometric mean)	No reduction	Meunier et al. [51]
**30**	Subunit vaccine with 240 µg of recombinant *Campylobacter* adhesion protein to fibronectin (CadF)^6^ mixed with MONTANIDE™ ISA 70 VG, intramuscularly with booster	*C. jejuni* F38011 (8.3) and Day 20	27	6.04 (median)	7.76 (median)	1.71 median log10 reductions ^5^	Neal-McKinney et al. [25]
**31**	Subunit vaccine with 240 µg recombinant FlaA^6^ mixed with MONTANIDE™ ISA 70 VG, intramuscularly with booster	*C. jejuni* F38011 (8.3) and Day 20	27	4.41 (median)	7.76 (median)	3.35 median log10 reductions ^5^	Neal-McKinney et al. [25]
**32**	Subunit vaccine with 240 µg recombinant fibronectin-like protein A (FlpA)^6^ mixed with MONTANIDE™ ISA 70 VG, intramuscularly with booster	*C. jejuni* F38011 (8.3) and Day 20	27	4.65 (median)	7.76 (median)	3.11 median log10 reductions ^5^	Neal-McKinney et al. [25]
**33**	Subunit vaccine with 240 µg recombinant a component of multidrug efflux pump (CmeC) ^6^ mixed with MONTANIDE™ ISA 70 VG, intramuscularly with booster	*C. jejuni* F38011 (8.3) and Day 20	27	6.39 (median)	7.76 (median)	No effect of reduction due to the widest range in the level of colonization observed by the authors from the original paper (even 1.37 median log10 reduction calculated from the supplement table provided ^5^)	Neal-McKinney et al. [25]
**34**	Subunit vaccine of 240 µg a fusion protein of recombinant CadF-FlaA-FlpA^7^ mixed with MONTANIDE™ ISA 70 VG, intramuscularly with booster	*C. jejuni* F38011 (8.3) and Day 20	27	4.6 (median)	7.76 (median)	3.16 median log10 reductions ^5^	Neal-McKinney et al. [25]
**35**	10^8^ cells of *E. coli* wzy::kan strain vectored vaccine expressing *C. jejuni* protein glycosylation (N-glycan), orally with booster	*C. jejuni* 81–176 (6.0) and Day 28	35	5.7 ^3^ (median) in Trial#1 7.6 ^3^ (median) in Trial#2	9.0 ^3^ (median) in Trial#1 9.6 ^3^ (median) in Trial#2	Significant 3.30 median log10 reductions in Trial#1 upon heterologous challenge Significant 2.00 median log10 reductions in Trial#2 upon heterologous challenge	Nothaft et al. [46]
**36**	A formalin-killed whole-cell vaccine with 6.75 × 10^7^ CFU of bacterins mixed with oil adjuvants, subcutaneously	*C. jejuni* PD-316 (5.0) and Day 72	128	6.8 ^3^	7.5 ^3^	Non-significant 0.7 log10 reduction ^5^	Okamura et al. [52]
**37**	A formalin-killed whole-cell vaccine with 6.75 × 10^7^ CFU of bacterins mixed with aluminum hydroxide gel adjuvant, subcutaneously with booster	*C. jejuni* PD-316 (5.0) and Day 72	128	6.7 ^3^	7.5 ^3^	Non-significant 0.8 log10 reduction ^5^	Okamura et al. [52]
**38**	Subunit vaccine with 40 µg of recombinant NHC flagellin mixed with 0.4 M urea, 10 mM Tris pH 9.0, 20% glycerol, 5 mM sucrose, *in ovo*	*C. jejuni* 81–116 (5.0) and Day 18	25	6.8 ^3^	7.3 ^3^	Non-significant 0.5 log10 reduction	Radomska et al. [53]
**39**	Subunit vaccine with 20 µg of recombinant NHC flagellin mixed with 0.4 M urea, 10 mM Tris pH 9.0, 20% glycerol, 5 mM sucrose, *in ovo*	*C. jejuni* 81–116 (5.0) and Day 18	25	7.3 ^3^	7.3 ^3^	No reduction	Radomska et al. [53]
**40**	10^7^ CFU of *Salmonella* Typhimurium (∆*aroA*) mutant-1 (STM-1) vectored vaccine expressing cysteine ABC transporter substrate-binding protein (CjaA) on chromosome	*C. jejuni* 81–116 (9.0) and Day 35	49	7.8 ^3^	8.9 ^3^	1.1 log10 reductions ^5^	Saxena et al. [24]
**41**	10^7^ CFU of STM-1 vectored vaccine expressing CjaA in PMW2 plasmids	*C. jejuni* 81–116 (9.0) and Day 35	49	7.5 ^3^	9.0 ^3^	1.5 log10 reductions ^5^	Saxena et al. [24]
**42**	10^7^ CFU of STM-1 vectored vaccine expressing glycoprotein Cj1496 periplasmic protein on chromosome	*C. jejuni* 81–116 (9.0) and Day 35	49	8.3 ^3^	8.9 ^3^	0.6 log10 reduction ^5^	Saxena et al. [24]
**43**	10^7^ CFU of STM-1 vectored vaccine expressing Cj1496 periplasmic protein in PMW2 plasmids	*C. jejuni* 81–116 (9.0) and Day 35	49	8.0 ^3^	9.0 ^3^	1.0 log10 reduction ^5^	Saxena et al. [24]
**44**	10^7^ CFU of STM-1 vectored vaccine expressing *Campylobacter* invasion antigen B (CiaB) on chromosome	*C. jejuni* 81–116 (9.0) and Day 35	49	7.7 ^3^	8.9 ^3^	1.2 log19 reductions ^5^	Saxena et al. [24]
**45**	10^7^ CFU of STM-1 vectored vaccine expressing CiaB in PMW2 plasmids	*C. jejuni* 81–116 (9.0) and Day 35	49	8.6 ^3^	9.0 ^3^	0.4 log10 reduction ^5^	Saxena et al. [24]
**46**	10^7^ CFU of STM-1 vectored vaccine expressing CadF on chromosome	*C. jejuni* 81–116 (9.0) and Day 35	49	7.8 ^3^	8.9 ^3^	1.1 log10 reductions ^5^	Saxena et al. [24]
**47**	10^7^ CFU of STM-1 vectored vaccine expressing CadF in PMW2 plasmids	*C. jejuni* 81–116 (9.0) and Day 35	49	7.5 ^3^	9.0 ^3^	1.5 log10 reductions ^5^	Saxena et al. [24]
**48**	10^7^ CFU of STM-1 vectored vaccine expressing CjaA, CadF, CiaB, and cj1496 on chromosome	*C. jejuni* 81–116 (9.0) and Day 35	49	7.0 ^3^	8.9 ^3^	1.9 log10 reductions ^5^	Saxena et al. [24]
**49**	10^7^ CFU of STM-1 vectored vaccine expressing CjaA, CadF, CiaB, and cj1496 in PMW2 plasmids	*C. jejuni* 81–116 (9.0) and Day 35	49	6.8 ^3^	9.0 ^3^	2.2 log10 reductions ^5^	Saxena et al. [24]
**50**	Subunit vaccine with 50 µg of recombinant hemolysin co-regulated protein (rHcp) mixed with Freund’s incomplete adjuvant, orally with booster	*C. jejuni* BCH 71 (8.0) and Day 28	35	6.9 ^3^	8.9 ^3^	Significant 0.5 log10 reduction	Singh et al. [54]
**51**	Subunit vaccine with 50 µg of rHcp entrapped in chitosan-Sodium tripolyphosphate nanoparticles (CS-TPP NPs) (CS-TPP-rhcp), orally with booster	*C. jejuni* BCH 71 (8.0) and Day 28	35	6.5 ^3^	7.53	Significant 1.0 log10 reduction (as reported in the original paper)	Singh et al. [54]
**52**	Cell lysate vaccine with 4.3 µg of *C. jejuni* cell lysates, orally	*C. jejuni* 81–176 (7.0) and Day 15	37	5.7 ^3^ in Trial#1 6.3 ^3^ in Trial#2	7.8 ^3^ in Trial#1 7.9 ^3^ in Trial#2	Significant 2.14 log10 reductions in Trial#1 (reported in the original paper) Significant 1.92 log10 reductions in Trial#2 (reported in the original paper)	Taha-Abdelaziz et al. [55]
**53**	Cell lysate vaccine with 21 µg of *C. jejuni* cell lysates, orally	*C. jejuni* 81–176 (7.0) and Day 15	37	6.9 ^3^	7.6 ^3^	Non-significant 0.7 log reduction	Taha-Abdelaziz et al. [55]
**54**	Cell lysate vaccine with 4.3 µg of *C. jejuni* cell lysates combined with 5 µg of E-CpG, orally	*C. jejuni* 81–176 (7.0) and Day 15	37	5.5 ^3^	7.9 ^3^ 6.9^3^	Significant 2.42 log10 reductions (compared with PBS as reported in the original paper) Significant 1.42 log10 reductions (compared with E-CpG alone) in this review as it was presented in the figure of the original paper	Taha-Abdelaziz et al. [55]
**55**	Subunit vaccine with 0.2 mg of recombinant DNA binding protein for biofilm formation (Dps) mixed with Freund’s complete adjuvant, subcutaneously with boosters	*C. jejuni* NCTC 11168 (5.0) and Day 34	44	8.12 (geometric mean)	7.96 (geometric mean)	No reduction	Theoret et al. [56]
**56**	Bacterial density (O.D.600 = 10.0, 0.50 mL) of *Salmonella* Typhimurium strain χ9088 vectored vaccine (OD600, 0.5 mL) expressing Dps, orally with boosters	*C. jejuni* NCTC 11168 (5.0) and Day 34	36	3.72 (geometric mean)	6.2 (geometric mean)	Significant 2.48 (geometric mean) log10 reductions	Theoret et al. [56]
**57**	2 × 10^10^ CFU of *Lactobacillus lactis* NZ3900 vectored vaccine expressing cysteine ABC transporter substrate-binding protein (CjaA) fused to heat-labile enterotoxin B subunit (LTB) of *E. coli* (CjaA-LT-B), orally with boosters	*C. jejuni* NCTC 11168 (6.2) and Day 33	42	6.8 ^3^	5.8 ^3^	No reduction	Wang et al. [57]
**58**	2 × 10^10^ CFU of *Lactobacillus lactis* NZ3900 vectored vaccine expressing CjaA, orally with boosters	*C. jejuni* NCTC 11168 (6.2) and Day 33	42	6.0 ^3^	5.8 ^3^	No reduction	Wang et al. [57]
**59**	Avirulent *Salmonella* Typhimurium χ3987 strain vectored vaccine (108 cells) expressing CjaA, orally with boosters	*C. jejuni* labeled with pUOA18 (8.3) and Day 28	40	<3.00 (below detection limit)	9.1 ^3^	Significant 6.0 log10 reductions (reported in the original paper) upon heterologous challenge	Wyszynska et al. [26]
**60**	10^8^ CFU of *Salmonella* Enteritidis (SE) vectored vaccine expressing Omp18 protein (Cj0013), peptidoglycan associated lipoprotein of *Salmonella* (PAL of *Salmonella*), and high mobility group box 1 protein (HMGB1), orally	*C. jejuni* field strain (6.8) and Day 7	43	7.14 ± 0.29	7.70 ± 0.29	Non-significant 0.56 log10 reduction	Yang et al. [58]
**61**	10^8^ CFU of SE vectored vaccine expressing HMGB1, PAL of *Salmonella*, and Cj0013, orally	*C. jejuni* field strain (6.8) and Day 7	43	7.5 ^3^	7.70 ± 0.29	Non-significant 0.2 log10 reduction (non-significant)	Yang et al. [58]
**62**	10^8^ CFU of SE vectored vaccine expressing Cj0013, HMGB1, and PAL of *Salmonella*, orally	*C. jejuni* field strain (6.8) and Day 7	43	7.6 ^3^	7.70 ± 0.29	Non-significant 0.1 log10 reduction	Yang et al. [58]

^1^ The arithmetic mean was the most commonly reported (mean *C. jejuni* loads in ceca) but some trials reported the geometric mean log10 or median log10 that are provided in in this table.; ^2^ Homologous challenge using the *C. jejuni* vaccine strain was commonly used in the trials.; ^3^ The value of mean log10 was estimated from the figures presented in the original papers.; ^4^ Broilers administered poly lactide-co-glycolide nanoparticles (NP) were the control group for the purposes of challenge as reported in the original paper.; ^5^ In these studies the non-vaccinated (broilers) groups (some injected with PBS) that were challenged, were considered the control groups in order to compare with the vaccinated groups; ^6^ Prime/boost vaccination regimen consisted of an antigen fused with Glutathione S-transferase tagged proteins (GST) prime followed by the antigen fused with polyhistidine tag proteins (HIS) in a booster vaccine; ^7^ Prime/boost vaccination regimen consisted of a combination of 80 µg CadF-GST, 80 µg FlaA-GST, and 80 µg FlpA-GST proteins in a prime followed by a combination of 80 µg CadF-His, 80 µg FlaA-His, and 80 µg FlpA-His proteins in a booster vaccine; GenEq/g, Genome equivalents per gram.

### 3.2. Vaccine Types

Overall, eight vaccine types (bacterial vector-based, subunit, DNA, a combination of vaccine, killed-whole cells, cell lysate, crude cell lysate, and conjugated vaccines) were identified in this review using the two definitions of vaccine efficacy. Based on the 62 trials with the *C. jejuni* loads in ceca of the vaccinated and non-vaccinated broilers investigated, the bacterial vector-based vaccines were the most frequently used in 19 trials from seven papers. Of these, *Salmonella* Typhimurium (ST) and *Salmonella* Enteritis (SE) vectors were used in 16 trials, followed by *Lactobacillus lactis* (*L. lactis*) (*n* = 2) and *Escherichia coli* (*E. coli*) (*n* = 1). Subunit vaccine (*n* = 12) and DNA (prime) with subunit (boost) vaccine (*n* = 12) were identified in six and two papers. Crude lysate vaccine was used in eight trials from one paper. DNA, Whole-cell lysate, and conjugated vaccines were used in nine trials from four papers. The remaining vaccine type used in two trials from one paper was a killed-whole cell vaccine.

Based on the 45 trials with the prevalence of colonized broilers reported, subunit vaccine was the most frequently used in 13 trials from seven papers, followed by a combination of DNA (prime) and subunit (boost) vaccines from 12 trials of two papers. The crude cell lysate vaccine was found in eight trials of one paper [47]. The bacterial vector-based vaccines used in six trials from three papers were *E. coli* wzy::kan strain [46], ST χ9088 strain [56], and Avirulent ST χ3987 strain [26]. While the use of whole-cell lysate (*n* = 3) and DNA (*n* = 3) vaccines were found in three papers.

### 3.3. Vaccine Antigens and Vaccine Regimens

The *C. jejuni* antigens evaluated in the vaccine efficacy studies included in this review are summarized in Table 4.

A total of 23 *C. jejuni* antigens used as single and/or multiple antigens in vaccine trials were identified using both terms of vaccine efficacy in this review. Based on the 62 trials with evaluations of *C. jejuni* loads reported, variations of antigens used in the trials, vaccine regimens, and the broiler age at the end of study (ranging from 25 to 128 days) were identified (Table 3). Of these, total outer membrane proteins (OMP) used in the crude lysate vaccine were the most frequent antigen evaluated after homologous challenge in 8 trials from one paper. This antigen was used either encapsulated with biodegradable and biocompatible poly (lactide-co-glycolide) nanoparticles (OMP-NP) or non-encapsulated via oral or subcutaneous vaccinations with a booster. Following this, cysteine ABC transporter substrate-binding protein (CjaA) used in five trials from three papers were evaluated in the oral vaccination with booster(s) using three different bacterial vectored vaccines: ST (∆*aroA*) mutant-1 (STM-1) (*n* = 3), *L. lactis* NZ3900 strain (*n* = 2) and, avirulent ST χ398 strain (*n* = 1). Of these five trials, the only vaccine trial using the avirulent ST χ398 strain expressing CjaA was challenged with heterologous *C. jejuni* strains. Flagellin A protein used in 4 trials (two papers) were evaluated three different vaccine types (DNA, subunit, and DNA (prime)/subunit (boost) vaccines) and routes of administrations (intramuscularly or subcutaneously with booster). Hypothetical protein YP99817.1 protein used in four trials was evaluated using three different vaccine types booster as well, but only one vaccinated route (intramuscularly with/without booster) was used and these trials were from only one paper. Three antigens (i.e., *Campylobacter* adhesion protein to fibronectin (CadF), whole-cell lysate, and capsular polysaccharide (CPS)) were used and evaluated upon homologous challenge in nine trials from four different papers. CadF identified in two papers was evaluated in subunit (intramuscularly with booster) and STM-1 vectored vaccines (orally with booster) after homologous challenge. Whole lysate and CPS were used in six trials from two papers. The whole-cell lysate was orally administrated with/without E-CpG, whereas CPS was conjugated with diphtheria toxoid of Corynebacterium diphtheriae vaccine (CPSconj) and mixed with 10 µg of CpG ODN 2007 for subcutaneous vaccination with a booster. The remaining trials using other antigens were less frequent studies (less than 2 trials) (Table 3).

Based on the prevalence of colonized broilers reported, several antigens were also identified in the 45 eligible trials conducted in various ages of broiler at the end of study ranging between 25 and 44 days old (Table 5). The antigens used in the 45 trials were a subset of the antigens used, based on the investigation of *C. jejuni* loads criteria except for an extra trial which was a subunit vaccine using recombinant flagellin A protein (FlaA) fused to heat-labile enterotoxin (LT-B) of *E.coli* (FlaA-LT-B) mixed with sodium carbonate, delivered orally with a booster (designated as Trial no. 63) in this review (Table 5). Of the 45 trials, the OMP (with/without NP) used in the crude lysate vaccine were the most common antigens used in eight trials, following this, FlaA (*n* = 5, three papers), hypothetical protein YP_999817.1 (*n* =4, one paper), whole-cell lysate (*n* = 3, one paper), and CjaA (*n* = 3, two papers). Seven antigens were used in 14 trials from five papers using different vaccine formulations and regimens (Table 5). These seven antigens were DNA binding protein for biofilm formation (Dps), flagellin, hemolysin co-regulated protein (Hcp), flagellin protein family (FlgL), *Campylobacter* hemolysin activation/secretion protein (YP_001000437.1), *C. jejuni* surface-exposed lipoprotein A (JlpA), and hypothetical protein YP99838.1. While the remaining eight trials were utilized eight different antigens (six individual and two multiple antigens). 

### 3.4. Levels of C. jejuni Loads (log10 CFU/g) in Cecal Contents as Vaccine Efficacy

The four different outcomes of vaccine efficacy reported from the 62 trials were identified in this review: no reduction (*n* = 9), log10 reductions (*n* = 15), non-significant log10 reductions (*n* = 20), and significant log10 reductions (*n* = 18). The four different reporting outcomes of log10 CFU/gram were also identified in this review (i.e., genome equivalents per reaction per gram, median log10 reductions, geometric log10 reduction, and arithmetic mean log10 reduction).

The 18 trials reported significant log10 reductions ranging between 0.5 log10 and 6.0 log10 of *C. jejuni* cecal loads upon homologous/heterologous challenge. Of these, an avirulent ST χ3987 strain vectored vaccine expressing CjaA (10^8^ CFU) administrated orally and a booster was the most significant levels of reductions (~6.0 log10) after heterologous challenge reported [26]. Following this, the crude cell lysate vaccine contained 125 µg of OMP or OMP-NP subcutaneously with booster (two trials) provided approximately 5.7 log10 reductions after homologous challenges, compared to the broiler vaccinated with NP alone estimated from the figure provided in the original paper [47]. Eight trials reported significant reduction levels of *C. jejuni* loads varied between 2.0 log10 and 4.27 (mean or median) log10 CFU/g after homologous/heterologous challenge. Of the eight trials, four trials were a combination of DNA (prime) with subunit (boost) vaccines using four antigens: *Campylobacter* hemolysin activation/secretion protein, FlaA, fibronectin-like protein A (FlpA), flagellin protein family (FlgL), hypothetical protein YP99838.1, or hypothetical protein YP99817.1, and a CadF-FlaA-FlpA) were from one paper [50]. Another four trials (from three papers). Another four trials were cell lysate vaccines using 4.3 µg of *C. jejuni* cell lysates with/without 5 µg of E-CpG (orally), *E. coli* wzy::kan strain vectored vaccine expressing *C. jejuni* protein glycosylation (N-glycan) (orally with a booster, Salmonella Typhimurium strain χ9088 vectored vaccine expressing DNA binding protein for biofilm formation (Dps) orally with a booster. The significant log10 reductions reported in seven trials were less than 1.0 log10 CFU/g.

Moreover, 15 trials from two papers reported levels of log10 CFU/g reductions without significant or non-significant reported. Of these, five trials with subunit vaccines contained recombinant CadF, FlaA, FlpA, a component of multidrug efflux pump (CmeC), a fusion protein of CadF-FlaA,-FlpA emulsified with MONTANIDE™ ISA70 VG intramuscularly with booster provide various reduction levels (between 1.37 and 3.16 median log10 reductions) and the original paper reported the subunit with CmeC did not prevent *C. jeuni* colonization after homologous challenge due to a wide range of *C. jejuni* loads in the individual vaccinated broilers [25]. Ten trials (from one paper) used STM-1 vectored vaccine expressing various antigens from the inserted plasmids or ST chromosome orally with booster reported the reduction levels between 0.4 log10 CFU/g and 2.2 log10 CFU/g after homologous challenge, estimated from the figures provided in the original paper [24]. 

Twenty trials from seven papers reported non-significant (geometric, arithmetic, or median) log10 reduction (CFU/g or GenEq/g) upon homologous/heterologous challenge. The levels of log10 reduction from 15 of 20 trials were reported between 0.1 and 1.6 (mean or median) log10 reduction after homologous challenge. These trials were the crude lysate vaccines with OMP, OMP-NP orally with booster [47], DNA vaccine with purified FlaA cloned into pcDNA3 plasmids mixed with adjuvant subcutaneously or intramuscularly with booster [51], formalin-killed whole-cell vaccine mixed with oil adjuvants [52], subunit vaccine with 40 µg of recombinant NHC flagellin mixed adjuvant in ovo [53], and SE vectored vaccine expressing Omp18 protein (Cj0013), peptidoglycan associated lipoprotein of *Salmonella* (PAL of *Salmonella*), and high mobility group box 1 protein (HMGB1) orally [58]. Five trial used a combination of DNA (prime) and subunit (boost) vaccine with four individual antigens and combinations of these antigens, delivered intramuscularly with booster upon heterologous challenge reported reduction levels ranging between 0.12 and 2.2 log10 CFU/g (using qPCR or bacterial culture methods) and between 1.06 and 1.92 log10 reductions in genome equivalents per gram (qPCR) [50].

Of further note, nine trials using hypothetical protein YP99817.1 in DNA (prime) and subunit (boost), 20 µg of recombinant flagellin-NHC mixed adjuvant in subunit vaccine in ovo, and *L. lactis* NZ3900 vectored vaccine expressing CjaA and heat-labile enterotoxin B subunit (LTB) of *E. coli* (CjaA-LTB) failed to reduce *C. jejuni* colonization [50,53,56,57].

### 3.5. Prevalence of Colonized Broilers in Vaccine Efficacy

To further evaluate vaccine performance in this review, the trial outputs from eligible studies were used to estimate vaccine efficacy using relative risks to enable comparisons to be made between studies. A wide range of vaccine efficacies in vaccinated broilers was identified, ranging from no effect of *C. jejuni* colonization to 100.0% prevention (Table 5). Of 45 eligible vaccine trials, three trials reported *C. jejuni* detection was unculturable (below detection limit) in all vaccinated broilers after homologous challenge (RR < 0.11) and vaccine efficacy was approximately 90%.The three trials were 125 µg of crude cell lysate vaccine with total OMP (subcutaneously with booster), 125 µg of crude cell lysate vaccine with total OMP encapsulated with lactide-co-glycolide nanoparticles (subcutaneously with booster), and 10^8^ cells of an avirulent Salmonella enterica χ3987 strain vectored vaccine expressing CjaA (orally with booster). Following these, one trial using a subunit vaccine with recombinant FlaA-LT-B mixed with sodium carbonate reported a significant reduction of the number of colonized vaccinated broilers after heterologous challenge with the prevalence of 27.6% [78] but the efficacy was 44% (Table 5).

In contrast, 28 trials failed to prevent *C. jejuni* colonization as all vaccinated broilers were positive of *C. jejuni* with a relative risk of ≥1.00 (Table 5). Based on a comparison of log10 CFU/g reductions, 10 trials report significant log10 reductions between 0.5 and 4.2 log10 CFU/g and 10 trials were non-significant log10 reductions ranging between 0.1 and 2.2 log10. While six and two trials were non-reduction and not reported, respectively (Table 5).

## 4. Discussion

The development of efficacious *C. jejuni* vaccines for poultry is potentially an effective intervention strategy to reduce the risk of campylobacter infections in humans. In this review, our goal was to evaluate the results of published *C. jejuni* vaccine studies with the view to identifying the most efficacious antigens for further development. The effective outcomes of controlling *C. jejuni* at farms are commonly evaluated using the reduction of prevalence (proportion) of colonized broilers or the reduction of *C. jejuni* loads in the intestine. When undertaking this review, it became apparent that the variability of how *C. jejuni* vaccine studies have been reported prevent direct comparisons of vaccine efficacy from being made. Most studies report the outcome of vaccination as either antigen-specific immune responses and/or reductions of the *C. jejuni* loads in the intestines [22,25,45,47,48,49,50,51,54,55,79,80,81,82]. While many studies report significant reductions in *C. jejuni* loads, the actual reductions are highly variable. Consequently, it is difficult to estimate the potential impacts of these studies on the risk of *C. jejuni* transmission to humans.

Based on this review, 62 trials from 16 papers fulfilled the selection criteria and were included using *C. jejuni* loads in vaccinated broilers [24,25,26,46,47,48,49,50,51,52,53,54,55,56,57,58]. The variations of *C. jejuni* log10 reduction within these studies were estimated from different *C. jejuni* loads (log10) in ceca between vaccinated and non-vaccinated chickens [49,50,80,82]. High variations of significant log10 reductions of *C. jejuni* loads in the intestines of vaccinated broilers were reported between 0.5 and 6.0 log10 reductions among the studies using different variables for statistical comparisons (i.e., geometric mean, arithmetic mean, or median) [25,26,49,50,54,55,56]. Highly variable data of *C. jejuni* loads in the individual vaccinated broilers were reported in some trials of the original papers was identified in this review [25,50]. In some cases, where levels of log10 reductions were identified, the outcomes of vaccine efficacy were reported as non-significant reduction or no decrease in *C. jejuni* colonization [25,50]. While other studies reported levels of CFU/g reduction of *C. jejuni* colonization between ~0.5 and ~1.9 were significant [49,54,55], other studies reported similar reductions (<1.9 log10 CFU/g) as non-significant [25,50,58]. These suggest that the statistical power of some studies was insufficient to discriminate between treatment groups where the log10 reductions of colonization were modest. Nauta et al. [10] estimated that a one or two log10 reduction of *Campylobacter* loads in cecal contents of broilers at slaughterhouses could potentially reduce the risk of transmission to humans by at least 44%. Therefore, more studies are needed to define the vaccine trial parameters required to enable the robust measurement of log10 reductions and how these reductions impact on the risk of human transmission. Defining these parameters is important as assessing the efficacy of *C. jejuni* vaccines as it is likely to remain reliant on challenge studies. Several studies have reported poor correlations between immune responses and reductions in the *C. jejuni* loads in the intestines of chickens in vaccination/challenge studies [22,25,45,47,50,51,54,79,81,82].

As a result of these factors, the quantitative risk assessment model reported by Rosenquist et al. [34] was adopted for this review. The model predicts that a 30-fold reduction in the broiler flock prevalence of *C. jejuni* would result in a 2-log10 reduction of carcass contamination. The outcome of reducing carcass contamination by this amount could result in a 30-fold decrease in the incidence of human campylobacteriosis. Similarly, EFSA [9] using a model for *C. jejuni* prevalence targets to analyse the quantitative microbiological risk assessment estimated that setting targets of 25% and 5% between broiler flock prevalence would reduce to 50% and 90% of the public health risk, respectively. Thus, these models enable the critical evaluation of published vaccine efficacy studies in the context of public health outcomes. Consequently, in this review, we used the proportionate number (prevalence) of *C. jejuni* positive/negative broiler chickens between vaccinated and unvaccinated after challenge to evaluate the included studies as another definition of vaccine efficacy. The prevalence of colonized broilers was taken from the text/tabulations reported and/or estimated from figures provided in the original papers. Consequently, a total of 45 trials from 13 papers fulfilled the inclusion criteria [25,26,46,47,48,50,51,53,54,55,56,57,78]. This highlights the need for future studies to consider the models of Rosenquist et al. [34] and EFSA [9] to determine the impact of reducing *C. jejuni* loads in ceca of chickens on the risk of carcass contamination. When considering the vaccine efficacy based upon prevalence, OMP, OMP-NP, and CjaA antigens from three different vaccine trials (crude cell lysate and avirulent ST χ3987 strain vectored vaccines) were demonstrated to clear *C. jejuni* colonization in the vaccinated broilers with RR < 0.11 and vaccine efficacy greater than 90%, compared with the control groups. These outcomes were comparable to significant levels between 5.7 and 6.0 log10 reductions reported. Following this, a subunit vaccine with 1 mg of recombinant FlaA-LT-B mixed with sodium carbonate reported significant reductions in the number of colonized broiler with prevalence of 27.59% [78], but the RR was 0.56 with the vaccine efficacy of 44%. Thus, based on the data reviewed using both definitions of vaccine efficacy, significant reduction levels more than 5.7 log10 reductions could provide the vaccine efficacy more than 90%.

One of the potential challenges for using vaccination to control *C. jejuni* colonization is the lifespan of commercial broilers. The current review identified that the many eligible vaccine efficacy studies used broilers with a wide age range, ranging from 24 to 46 days by the end of the study (Table 3 and Table 5). Commercial broiler chickens are commonly slaughtered between 35 and 86 days of age, depending on the target market weight and the type of farming system [83,84]. It has been reported that chicken B cell populations do not fully mature until 42 days of age, which may also affect vaccine efficacy [85]. Chicken age is of further importance to vaccine efficacy with respect to timing of *C. jejuni* colonization. Recent studies have reported that commercial broilers were colonized by *C. jejuni* and/or *C. coli* by 10 days of age [86,87], suggesting that vaccination of chicks would be of benefit to the poultry industry. However, maternal antibodies can interfere with vaccine efficacy when live vectored vaccines are applied in young chicks [88]. To overcome this issue, a subunit vaccine or a vectored vaccine with various routes of immunization (i.e., intranasal or *in ovo*) that are not neutralized by maternal antibodies would be worthwhile exploring [89,90,91]. Thus, the ideal *C. jejuni* vaccine will need to confer rapid immune responses to antigens associated with preventing colonization and provide protection to chickens from early in the production cycle through to slaughter.

Based upon the inclusion/exclusion criteria of this review using the prevalence of colonized broilers, a meta-analysis could not be performed due to highly variable data. Thus, it is recommended that future studies reporting *C. jejuni* efficacy studies are supported by datasets that include, the numbers of colonized/non-colonized broiler chickens in treatment groups. Where the outcomes of trials are reported as a degree of colonization (e.g., CFU/g of fecal matter) individual chicken data should be reported to enable future meta-analyses of vaccine studies.

The compiled dataset of published *C. jejuni* poultry vaccine studies reviewed here has highlighted the highly variable nature of how these prototype vaccines have been evaluated and reported. However, it is clear from the results of these vaccine studies, some of these could potentially lead to a commercial vaccine in the future. Thus, it is recommended that a standardized evaluation model and reporting system be developed for *C. jejuni* vaccination studies. The standardized evaluation model would need to include, bird type (e.g., broiler and layer), age of bird, type of vaccine, antigen (source and dose), type of adjuvant where applicable, route of vaccination, method of challenge, time to challenge, and challenge dose(s) being the minimal reporting requirements. In terms of evaluating efficacy, while various outcomes would be acceptable, such as protected/not protected or reductions in colonization loads, based on bacterial culture and/or molecular (i.e., quantitative PCR or mass spectrometry) detection, it is crucial that individual bird data should be made readily available. Standardization, particularly of efficacy trial outcome reporting, would enable a more robust evaluation of putative antigens and their formulations between studies.

## 5. Conclusions

Of the *C. jejuni* antigens evaluated in this study, it was concluded that the OMP (125 µg) formulated with and without PLGA-NP delivered subcutaneously and the oral vaccination with subunit vaccine with recombinant FlaA-LT-B mixed with sodium carbonate were the most efficacious candidate vaccines to reduce *C. jejuni* colonization of broilers identified to date. Further evaluation of this “antigen complex” is clearly warranted, perhaps using OMP preparations from gene deletion mutants to identify which components are contributing to the protection, using the proposed evaluation model described above. Overall, the data assessed in this review supports the conclusion that the development of a *C. jejuni* vaccine to prevent the colonization of poultry is feasible. Such a vaccine would be crucial in helping the global poultry industry minimize risks to the consumers of their products.

## Figures and Tables

**Table 1:** The algorithm of systematic search terminology.

Database	Term Search Outcome
**PubMed Central**	“Campylobacter”[All Fields] AND “Vaccine”[All Fields] AND “Chicken”[All Fields] AND (“1970/01/01”[PDat]: “2020/12/31”[PDat])
**Elsevier ScienceDirect**	“Campylobacter” AND “Vaccine” AND “Chicken”
**Scopus**	TITLE-ABS-KEY (“Campylobacter” AND “Vaccine” AND “Chicken”) AND (LIMIT-TO (PUBYEAR, 2020) OR LIMIT-TO (PUBYEAR, 2019) OR LIMIT-TO (PUBYEAR, 2018) OR LIMIT-TO (PUBYEAR, 2017) OR LIMIT-TO (PUBYEAR, 2016) OR LIMIT-TO (PUBYEAR, 2015) OR LIMIT-TO (PUBYEAR, 2014) OR LIMIT-TO (PUBYEAR, 2013) OR LIMIT-TO (PUBYEAR, 2012) OR LIMIT-TO (PUBYEAR, 2010) OR LIMIT-TO (PUBYEAR, 2009) OR LIMIT-TO (PUBYEAR, 2008) OR LIMIT-TO (PUBYEAR, 2007) OR LIMIT-TO (PUBYEAR, 2006) OR LIMIT-TO (PUBYEAR, 2005) OR LIMIT-TO (PUBYEAR, 2004) OR LIMIT-TO (PUBYEAR, 2003) OR LIMIT-TO (PUBYEAR, 2002) OR LIMIT-TO (PUBYEAR, 2001) OR LIMIT-TO (PUBYEAR, 2000) OR LIMIT-TO (PUBYEAR, 1999) OR LIMIT-TO (PUBYEAR, 1998) OR LIMIT-TO (PUBYEAR, 1997) OR LIMIT-TO (PUBYEAR, 1996) OR LIMIT-TO (PUBYEAR, 1995) OR LIMIT-TO (PUBYEAR, 1994) OR LIMIT-TO (PUBYEAR, 1993) OR LIMIT-TO (PUBYEAR, 1992) OR LIMIT-TO (PUBYEAR, 1991) OR LIMIT-TO (PUBYEAR, 1990) OR LIMIT-TO (PUBYEAR, 1989) OR LIMIT-TO (PUBYEAR, 1988) OR LIMIT-TO (PUBYEAR, 1987) OR LIMIT-TO (PUBYEAR, 1986) OR LIMIT-TO (PUBYEAR, 1985) OR LIMIT-TO (PUBYEAR, 1984) OR LIMIT-TO (PUBYEAR, 1983) OR LIMIT-TO (PUBYEAR, 1982) OR LIMIT-TO (PUBYEAR, 1981) OR LIMIT-TO (PUBYEAR, 1980) OR LIMIT-TO (PUBYEAR, 1979) OR LIMIT-TO (PUBYEAR, 1978) OR LIMIT-TO (PUBYEAR, 1977) OR LIMIT-TO (PUBYEAR, 1976) OR LIMIT-TO (PUBYEAR, 1975) OR LIMIT-TO (PUBYEAR, 1974) OR LIMIT-TO (PUBYEAR, 1973) OR LIMIT-TO (PUBYEAR, 1972) OR LIMIT-TO (PUBYEAR, 1971) OR LIMIT-TO (PUBYEAR, 1970))

**Table 2:** Inclusion and exclusion criteria in this study.

Process	Inclusion Criteria	Exclusion Criteria
**Screening**	**Primary:** 1. Vaccine studies conducted in chickens 2. Primary research studies containing vaccinated and unvaccinated groups ^1^ 3. Information of vaccines and vaccination protocols provided (vaccine formulas, antigen candidate, vaccine dosage, number of vaccination, route of vaccine administration, age of chickens or embryonic eggs when vaccination, challenge strain, age of chickens at the challenge, sample size, and chicken breed) 4. Evaluation and data of vaccine efficacy provided 5. English language	**Primary:** 1. Review articles and guidelines 2. Non-vaccine studies, non-challenge studies, or in vitro studies 3. Non-chicken model studies 4. Non-*Campylobacter* vaccine studies 5. Non-English language 6. No author name provided 7. Unable to access the full text of papers
**Eligibility**	**Secondary:** 1. Vaccine studies conducted in broiler chickens 2. Studies described the levels of *C. jejuni* loads in cecal contents (log10 CFU/gram or CFU/gram) and/or numbers of the individual (colonized ^2^ and non-colonized ^3^) broiler chickens after vaccinations and challenge	**Secondary:** 1. Vaccines conducted in layer chickens 2. Co-administration studies other than vaccine studies 2. Studies evaluated immune response alone without an effect of *C. jejuni* colonization after challenge 3. Studies evaluated the adjuvant efficacy alone or non-*C. jejuni* antigens 4. Studies evaluated vaccine efficacy using samples other than ceca (i.e., ileum and cloaca) 5. Studies conducted in some challenged chickens after vaccination (defined as a seeder-bird colonization model) 6. Studies that were unable to estimate *C. jejuni* loads and/or the number of colonized broilers from figures

^1^ The non-vaccinated (control) groups were defined as the groups of chickens that were conducted in the same vaccine regimen, compared with the vaccinated groups, but administrated with placebo, adjuvants only, parent vectored vaccines, or parent plasmids (without any inserts); ^2^ Colonized chickens in this study were defined by detecting or enumerating *C. jejuni* from cecal samples collected using assays in the original studies. ^3^ Non-colonized chickens were defined when the *C. jejuni* was unculturable or below the detection level of the assays in study samples.

**Table 4:** Summary of *Campylobacter jejuni* vaccine antigens identified in this review.

Vaccine Antigen	Role of Antigen in Promoting *C. jejuni* Colonization of Host	Reference
**Bacterin**	Killed-whole bacterial cells (multiple antigens) used for immunization	Okamura et al. [52]
***C. jejuni* glycoprotein Cj1496**	Invasion	Kakuda and DiRita [59]
***Campylobacter* adhesion protein to fibronectin (CadF)**	Adhesion	Konkel et al. [60]
***Campylobacter* invasion antigen B (CiaB)**	Invasion	Konkel [61]
***Campylobacter* surface-exposed lipoprotein A (JlpA)**	Adhesion	Jin et al. [62]
**Capsular polysaccharide (CPS)**	Serum resistance	Keo et al. [63]
**Component of multidrug efflux pump (CmeC)**	Multidrug efflux system	Lin et al. [64]
**Cysteine ABC transporter substrate-binding protein (CjaA)**	*Campylobacter* solute-binding protein and a component of the ABC transport system	Muller et al. [65]
**DNA binding protein for biofilm formation (Dps)**	Biofilm formation	Theoret et al. [56]
**Fibronectin-like protein A (FlpA)**	Adhesion	Konkel et al. [66]
**Flagellar hook-associated protein (FlgK)**	Motility	Fernando et al., [67] and Neal-McKinney and Konkel [68]
**Flagellar hook-basal body complex protein (FlgE-1)**	Motility and deliver *Campylobacter* invasion antigens (Cia proteins) to host cells	Neal-McKinney and Konkel [68]
**Flagellin**	Motility	Nachamkin et al. [69]
**Flagellin A protein (FlaA)**	Motility, adherence, and invasion	Wassenaar et al. [70]
**Flagellin protein family (FlgL)**	Deliver *Campylobacter* invasion antigens (Cia proteins) to host cells	Neal-McKinney and Konkel [68]
**Hemolysin co-regulated protein (Hcp)**	Secretion tube and effector protein in *Campylobacter jejuni* Type VI secretion system (T6SS) for adhesion and invasion	Liaw et al. [71] and Lertpiriyapong et al. [72]
**Hypothetical protein (YP_999817.1)**	Not fully described	Meunier et al. [50]
**Hypothetical protein (YP_999838.1)**	Protein-protein interactions	Meunier et al. [50]
**N-linked protein glycosylation (N-glycan)**	Protect *C. jejuni* surface proteins from gut protease and attachment to host cells	Alemka et al. [73] and Karlyshev et al. [74]
**Outer membrane proteins**	Adhesion and invasion	Chart et al. [75]
**Peptidoglycan-associated essential protein (PAL; Omp18; CjaD)**	Maintenance cell wall	Godlewska et al. [76]
**Whole-cell lysate**	Adhesion and invasion	Konkel and Joens [77]
**YP_001000437.1**	Activation/secretion of hemolysin	Meunier et al. [50]

**Table 5:** Summary of the vaccine efficacy from the eligible trials based on the prevalence of colonized broilers at the end of study.

Trial No	Vaccine Formulations, Antigens, and Regimens	Age of Chickens at the End of Study (Days)	Reductions of *C. jejuni* (log10) Colonization in Cecal Contents after Challenge ^1^		% Colonized Broilers in the Vaccinated Group (Proportion)	% Colonized Broilers in the Control Group (Proportion)	Relative Risk ^6^ (95% CI)	Efficacy (%) ^7^ against Colonization	Reference
			Significant (Yes/No)	Reduction Levels (Mean log10 CFU/gram ^2^) Reported					
**1**	Crude cell lysate vaccine with 125 µg of total outer membrane proteins (OMP) encapsulated with ploy lactide-co-glycolide nanoparticles (OMP-NP), orally with booster	42	No	0.4 log 10 reduction ^3^	87.5 (7/8)	57.1 (4/7)	1.53 (0.76 and 3.06)	No effect	[47]
**2**	125 µg of crude cell lysate vaccine with OMP, orally with booster	42	No	0.8 log10 reduction ^3,4^	62.5 (5/8)	57.1 (4/7) ^4^	1.09 (0.47 and 2.52)	No effect	[47]
**3**	125 µg of crude cell lysate vaccine with OMPs-NP, subcutaneously with booster	42	Yes	5.7 log 10 reductions ^3^	0.0 (0/8)	57.1 (4/7)	0.10 (0.01 and 1.56)	90	[47]
**4**	125 µg of crude cell lysate vaccine with OMP, orally with booster	42	Yes	5.7 log 10 reductions^3,4^	0.0 (0/8)	57.1 (4/7) ^4^	0.10 (0.01 and 1.56)	90	[47]
**5**	25 µg of crude cell lysate vaccine with OMP-NP, subcutaneously with booster	42	No	1.2 log 10 reductions ^3^	62.5 (5/8)	57.1 (4/7)	1.09 (0.47 and 2.52)	No effect	[47]
**6**	250 µg of crude cell lysate vaccine with OMP-NP, orally with booster	42	No	0.9 log 10 reductions ^3^	37.5 (3/8)	57.1 (4/7)	0.66 (0.22 and 1.97)	34	[47]
**7**	25 µg of crude cell lysate vaccine with OMP, orally with booster	42	No	1.6 log 10 reductions^3,4^	66.7 (NI)	57.1 (4/7) ^4^	Unable to calculate	Unable to calculate	[47]
**8**	250 µg of crude cell lysate vaccine with OMP, orally with booster	42	No	1.0 log 10 reduction ^3,4^	50.0 (4/8)	57.1 (4/7) ^4^	0.88 (0.34 and 2.25)	13	[47]
**9**	10^9^ CFU of *L. lactis* NZ9000 strain vectored vaccine expressing *C. jejuni* surface-exposed lipoprotein A (JlpA), intragastrically with booster	35	Yes Yes Yes	0.79 log10 reduction in Trial#1 0.47 log10 reduction in Trial#2 0.482 log10 reduction in Trial#3	100.0 (15/15)	100.0 (15/15)	1.00 (1.00 and 1.00)	No effect	[48]
**10**	Subunit vaccine with 50 µg of recombinant JlpA emulsified in Freund’s incomplete adjuvant, subcutaneously with booster	35	NoYesYes	0.11 log10 in Trial#1 0.69 log10 in Trial#2 0.49 log10 in Trial#3	100.0 (15/15)	100.0 (15/15)	1.00 (1.00 and 1.00)	No effect	[48]
**14**	DNA vaccine (prime) with 300 µg of purified DNA of *Campylobacter* hemolysin activation/secretion protein (YP_001000437.1) cloned into pcDNA3 plasmids mixed with 50 µg of CpG ODN2007 and subunit vaccine (boost) with 100 µg of recombinant YP_001000437.1 protein emulsified with MONTANIDE™ ISA70 VG, intramuscularly with booster	42	Yes	3.61 log10 reduction upon heterologous challenge	87.5 (14/16)	100.0 (15/15)	0.88 (0.73 and 1.05)	13	[50]
**15**	DNA vaccine (prime) with 300 µg of purified DNA of YP_001000437.1 cloned into pcDNA3 plasmids mixed with 50 µg of CpG ODN2007 and subunit vaccine (boost) with 100 µg of recombinant YP_001000437.1 protein emulsified with MONTANIDE™ ISA70 VG, intramuscularly with booster	42	No	1.92 log10 GenEq/g reductions upon heterologous challenge	80.0 (12/15)	93.8 (15/16)	0.85 (0.64 and 1.13)	15	[50]
**16**	DNA vaccine (prime) with 300 µg of purified DNA of flagellin protein family (FlgL) cloned into pcDNA3 plasmids mixed with 50 µg of CpG ODN2007 and subunit vaccine (boost) with 100 µg of recombinant FlgL emulsified with MONTANIDE™ ISA70 VG, intramuscularly with booster	42	Yes	2.03 log10 reductions upon heterologous challenge	100.0 (15/15)	100.0 (15/15)	1.00 (1.00 and 1.00)	No effect	[50]
**17**	DNA vaccine (prime) with 300 µg of purified DNA of FlgL cloned into pcDNA3 plasmids mixed with 50 µg of CpG ODN2007 and subunit vaccine (boost) with 100 µg of recombinant FlgL emulsified with MONTANIDE™ ISA70 VG, intramuscularly with booster	42	No	1.06 log10 GenEq/g reductions upon heterologous challenge	75.0 (12/16)	93.8 (15/16)	0.80 (0.59 and 1.09)	20	[50]
**18**	DNA vaccine (prime) with 300 µg of purified DNA of hypothetical protein (YP99838.1) cloned into pcDNA3 plasmids mixed with 50 µg of CpG ODN2007 and subunit vaccine (boost) with 100 µg of recombinant YP99838.1 emulsified with MONTANIDE™ ISA70 VG, intramuscularly with booster	42	Yes	2.08 log10 reductions upon heterologous challenge	100.0 (14/14)	100.0 (15/15)	1.00 (1.00 and 1.00)	No effect	[50]
**19**	DNA vaccine (prime) with 300 µg of purified DNA of YP99838.1 cloned into pcDNA3 plasmids mixed with 50 µg of CpG ODN2007 and subunit vaccine (boost) with 100 µg of YP99838.1 emulsified with MONTANIDE™ ISA70 VG, intramuscularly with booster	42	No	No reduction upon heterologous challenge	100.0 (14/14)	93.8 (15/16)	1.07 (0.94 and 1.21)	No effect	[50]
**20**	DNA vaccine (prime) with 300 µg of purified DNA of hypothetical protein (YP99817.1) cloned into pcDNA3 plasmids mixed with 50 µg of CpG ODN2007 and subunit vaccine (boost) with 100 µg of recombinant YP99817.1 emulsified with MONTANIDE™ ISA70 VG, intramuscularly with booster	42	Yes	4.27 log10 reductions upon heterologous challenge	92.3 (12/13)	100.0 (15/15)	0.92 (0.79 and 1.08)	8	[50]
**21**	DNA vaccine (prime) with 300 µg of purified DNA of YP99817.1 cloned into pcDNA3 plasmids mixed with 50 µg of CpG ODN2007 and subunit vaccine (boost) with 100 µg of recombinant YP99817.1 emulsified with MONTANIDE™ ISA70 VG, intramuscularly with booster	42	No	No reduction upon heterologous challenge	93.8 (15/16)	93.8 (15/16)	1.00 (0.84 and 1.20)	No effect	[50]
**22**	DNA vaccine with 300 µg of purified DNA of YP99817.1 cloned into pcDNA3 plasmids mixed with 50 µg of CpG ODN2007, intramuscularly with booster	42	No	No reduction upon heterologous challenge	100.0 (15/15)	100.0 (15/15)	1.00 (1.00 and 1.00)	No effect	[50]
**23**	Subunit vaccine with 100 µg of recombinant YP99817.1 emulsified with MONTANIDE™ ISA70 VG, intramuscularly with booster	42	No	No reduction upon heterologous challenge	100.0 (15/15)	100.0 (15/15)	1.00 (1.00 and 1.00)	No effect	[50]
**24**	DNA vaccine (prime) with 300 µg of purified DNA of flagellar hook-basal body complex protein (FlgE-1) cloned into pcDNA3 plasmids mixed with 50 µg of CpG ODN2007 and subunit vaccine (boost) with 100 µg of recombinant FlgE-1 emulsified with MONTANIDE™ ISA70 VG, intramuscularly with booster	42	No	2.20 log10 reductions a wide range of individual colonized broilers was presented in Figure 2A of the original paper upon heterologous challenge	91.7 (11/12)	100.0 (15/15)	0.92 (0.77 and 1.09)	8	[50]
**25**	DNA vaccine (prime) with 300 µg of purified DNA of flagellar hook-associated protein (FlgK) cloned into pcDNA3 plasmids mixed with 50 µg of CpG ODN2007 and subunit vaccine (boost) with 100 µg of recombinant FlgK emulsified with MONTANIDE™ ISA70 VG, intramuscularly with booster	42	No	1.72 log10 reductions but a wide range of individual colonized broilers was presented in Figure 2A of the original paper upon heterologous challenge	100.0 (14/14)	100.0 (15/15)	1.00 (1.00 and 1.00)	No effect	[50]
**26**	DNA vaccine (prime) with 300 µg of multiple DNA proteins (a combination of purified YP_001000437.1, FlgL, FlgK, FliE-1, YP99817.1, and YP99838.1) cloned into pcDNA3 plasmids mixed with 50 µg of CpG ODN2007 and subunit vaccine (boost) with 100 µg of recombinant multiple proteins (YP_001000437.1, FlgL, FlgK, FliE-1, YP99817.1, and YP99838.1) emulsified with MONTANIDE™ ISA70 VG, intramuscularly with booster	42	No	0.12 log10 reduction (No decrease of *C. jejuni* colonization reported in the original paper) upon heterologous challenge	100.0 (9/9)	100.0 (15/15)	1.00 (1.00 and 1.00)	No effect	[50]
**27**	DNA vaccine with 100 µg of purified DNA of flagellin A protein (FlaA) cloned into pcDNA3 plasmid mixed with 25 µg of CpG ODN2007, subcutaneously with booster	42	No	0.1 geometric mean log10 reduction ^3^	100.0 (15/15)	100.0 (15/15)	1.15 (0.95 and 1.41)	No effect	[51]
**28**	DNA vaccine with 100 µg of purified DNA of FlaA cloned into pcDNA3 plasmid mixed with 25 µg of CpG ODN2007, intramuscularly with booster	42	No	0.2 median log10 reductions^3^	75.0 (12/16)	87.5 (14/16)	0.86 (0.61 and 1.20)	14	[51]
**29**	DNA vaccine (prime) with 150 µg of purified DNA of FlaA into pcDNA3 plasmid mixed with 25 µg of CpG ODN2007 and subunit vaccine (boost) with 100 µg of recombinant FlaA emulsified with MONTANIDE™ ISA70 VG, intramuscularly with booster	42	No	No reduction	100.0 (16/16)	87.5 (14/16)	1.14 (0.95 and 1.38)	No effect	[51]
**30**	Subunit vaccine with 240 µg of recombinant *Campylobacter* adhesion protein to fibronectin (CadF) ^8^ mixed with MONTANIDE™ ISA 70 VG, intramuscularly with booster	27	Not reported	1.71 median log10 reductions ^5^	100.0 (11/11)	100.0 (12/12)	1.00 (1.00 and 1.00)	No effect	[25]
**31**	Subunit vaccine with 240 µg recombinant FlaA ^8^ mixed with MONTANIDE™ ISA 70 VG, intramuscularly with booster	27	Not reported	3.35 median log10 reductions ^5^	91.7 (11/12)	100.0 (12/12)	0.92 (0.77 and 1.09)	8	[25]
**32**	Subunit vaccine with 240 µg recombinant fibronectin-like protein A (FlpA1) mixed with MONTANIDE™ ISA 70 VG, intramuscularly with booster	27	Not reported	3.11 median log10 reductions ^5^	90.0 (9/10)	100.0 (12/12)	0.90 (0.73 and 1.11)	10	[25]
**33**	Subunit vaccine with 240 µg recombinant a component of multidrug efflux pump (CmeC) ^8^ mixed with MONTANIDE™ ISA 70 VG, intramuscularly with booster	27	Not reported	No effect of reduction due to the widest range in the level of colonization observed by the authors from the original paper (even 1.37 median log10 reduction calculated from the supplement table provided ^5^)	100.0 (12/12)	100.0 (12/12)	1.00 (1.00 and 1.00)	No effect	[25]
**34**	Subunit vaccine of 240 µg a fusion protein of recombinant CadF-FlaA-FlpA ^9^ mixed with MONTANIDE™ ISA 70 VG, intramuscularly with booster	27	Not reported	3.16 median log10 reductions ^5^	77.8 (7/9)	100.0 (12/12)	0.78 (0.55 and 1.10)	22	[25]
**35**	10^8^ cells of *E. coli* wzy::kan strain vectored vaccine expressing *C. jejuni* protein glycosylation (N-glycan), orally with booster	35	Yes Yes	3.30 median log10 reductions^3^ in Trial#1 upon heterologous challenge 2.00 median log10 reductions ^3^ in Trial#2 upon heterologous challenge	60.0 (9/15)	100.0 (15/15)	0.60 (0.40 and 0.91)	40	[46]
**38**	Subunit vaccine with 40 µg of recombinant NHC flagellin mixed with 0.4 M urea, 10 mM Tris pH 9.0, 20% glycerol, 5 mM sucrose, *in ovo*	25	No	0.5 log10 reduction ^3^	88.9 (8/9)	90.0 (9/10)	0.99 (0.72 and 1.35)	1	[53]
**39**	Subunit vaccine with 20 µg of recombinant NHC flagellin mixed with 0.4 M urea, 10 mM Tris pH 9.0, 20% glycerol, 5 mM sucrose, *in ovo*	25	No	No reduction	90.0 (9/10)	90.0 (9/10)	1.00 (0.75 and 1.34)	No effect	[53]
**50**	Subunit vaccine with 50 µg of recombinant hemolysin co-regulated protein (rHcp) mixed with Freund’s incomplete adjuvant, orally with booster	35	Yes	0.5 log10 reduction	100.0 (12/12)	100.0 (12/12)	1.00 (1.00 and 1.00)	No effect	[54]
**51**	Subunit vaccine with 50 µg of rHcp entrapped in chitosan-Sodium tripolyphosphate nanoparticles (CS-TPP NPs) (CS-TPP-rhcp), orally with booster	35	Yes	1.0 log10 reduction	100.0 (12/12)	100.0 (12/12)	1.00 (1.00 and 1.00)	No effect	[54]
**52**	Cell lysate vaccine with 4.3 µg of *C. jejuni* cell lysates, orally	37	Yes Yes	2.14 log10 reductions in Trial#1 1.92 log10 reductions in Trial#2	100.0 (20/20)	100.0 (19/19)	1.00 (1.00 and 1.00)	No effect	[55]
**53**	Cell lysate vaccine with 21 µg of *C. jejuni* cell lysates, orally	37	No	0.7 log reduction ^3^	100.0 (10/10)	100.0 (9/9)	1.00 (1.00 and 1.00)	No effect	[55]
**54**	Cell lysate vaccine with 4.3 µg of *C. jejuni* cell lysates combined with 5 µg of E-CpG, orally	37	Yes No	2.42 log10 reductions (compared with PBS as reported in the original paper) 1.42 log10 reductions ^3^ (compared with E-CpG alone) in this review as it was presented in the figure of the original paper	100.0 (9/9)	100.0 (10/10)	1.00 (1.00 and 1.00)	No effect	[55]
**55**	Subunit vaccine with 0.2 mg of recombinant DNA binding protein for biofilm formation (Dps) mixed with Freund’s complete adjuvant, subcutaneously with boosters	44	No	No reduction	100.0 (13/13)	100.0 (12/12)	1.00 (1.00 and 1.00)	No effect	[56]
**56**	*Salmonella* Typhimurium strain χ9088 vectored vaccine (OD600, 0.5 mL) expressing Dps, orally with boosters	36	Yes	2.48 (geometric mean) log10 reductions	100.0 (14/14)	100.0 (10/10)	1.00 (1.00 and 1.00)	No effect	[56]
**57**	2 × 10^10^ CFU of *Lactobacillus lactis* NZ3900 vectored vaccine expressing cysteine ABC transporter substrate-binding protein (CjaA) fused to heat-labile enterotoxin B subunit (LTB) of *E. coli* (CjaA-LT-B), orally with boosters	42	No	No reduction	100.0 (6/6)	100.0 (6/6)	1.00 (1.00 and 1.00)	No effect	[57]
**58**	2 × 10^10^ CFU of *Lactobacillus lactis* NZ3900 vectored vaccine expressing CjaA, orally with boosters	42	No	No reduction	100.0 (6/6)	100.0 (6/6)	1.00 (1.00 and 1.00)	No effect	[57]
**59**	10^8^ cells of avirulent *Salmonella* Typhimurium χ3987 strain vectored vaccine (10^8^ cells) expressing CjaA, orally with boosters	40	Yes	6.0 log10 reductions (reported in the original paper) upon heterologous challenge	0.0 (0/4)	100.0 (3/3)	0.11 (0.01 and 1.63)	89	[26]
**63**	Subunit vaccine with 1 mg of CjaA-LT-B mixed with sodium carbonate, orally with booster	35	No reported	Not reported	27.6 (40/145)	49.3 (70/142)	0.56 (0.41 and 0.76)	44	[78]

^1^ Homologous challenge using the *C. jejuni* vaccine strain was commonly used in the trials.; ^2^ The arithmetic mean was the most commonly reported (mean *C. jejuni* loads in ceca) but some trials reported the geometric mean log10 or median log10 that are provided in in this table.; ^3^ The value of mean log10 was estimated from the figures presented in the original papers.; ^4^ Broilers administered poly lactide-co-glycolide nanoparticles (NP) were the control group for the purposes of challenge as reported in the original paper.; ^5^ In these studies the non-vaccinated (broilers) groups (some injected with PBS) that were challenged, were considered the control groups in order to compare with the vaccinated groups.; ^6^ Relative risk was calculated in the current review based on the data provided in the original papers; ^7^ Efficacy estimate = (1 − RR) × 100 [39,41,42]; ^8^ Prime/boost vaccination regimen consisted of an antigen fused with Glutathione S-transferase tagged proteins (GST) prime followed by the antigen fused with polyhistidine tag proteins (HIS) in a booster vaccine.; ^9^ Prime/boost vaccination regimen consisted of a combination of 80 µg CadF-GST, 80 µg FlaA-GST, and 80 µg FlpA-GST proteins in a prime followed by a combination of 80 µg CadF-His, 80 µg FlaA-His, and 80 µg FlpA-His proteins in a booster vaccine.; CI, Confidence Interval; GenEq/g, Genome equivalents per gram; NI, Not identified as the percentage of colonized broilers reported in the original paper was not related to the number of colonized broilers provided in the same paper.

## Data Availability

The primary datasets extracted from the manuscripts included in this review were tabulated and summarized in Microsoft Excel. These files are available upon request to the corresponding author.

## References

[1] Kaakoush N.O., Castaño-Rodríguez N., Mitchell H.M., Man S.M. (2015). Global Epidemiology of Campylobacter Infection. Clin. Microbiol. Rev..

[2] Igwaran A., Okoh A.I. (2019). Human campylobacteriosis: A public health concern of global importance. Heliyon.

[3] (2016). EFSA, The European Union summary report on trends and sources of zoonoses, zoonotic agents and food-borne outbreaks in 2015. EFSA J..

[4] Moffatt C.R., Fearnley E., Bell R., Wright R., Gregory J., Sloan-Gardner T., Kirk M., Stafford R. (2020). Characteristics of Campylobacter Gastroenteritis Outbreaks in Australia, 2001 to 2016. Foodborne Pathog. Dis..

[5] (2010). EFSA, Scientific Opinion on Quantification of the risk posed by broiler meat to human campylobacteriosis in the EU. EFSA J..

[6] O’Leary M.C., Harding O., Fisher L., Cowden J. (2008). A continuous common-source outbreak of campylobacteriosis associated with changes to the preparation of chicken liver pâté. Epidemiol. Infect..

[7] Meade K.G., Narciandi F., Cahalane S., Reiman C., Allan B., O’Farrelly C. (2008). Comparative in vivo infection models yield insights on early host immune response to Campylobacter in chickens. Immunogenetics.

[8] Romero-Barrios P., Hempen M., Messens W., Stella P., Hugas M. (2013). Quantitative microbiological risk assessment (QMRA) of food-borne zoonoses at the European level. Food Control..

[9] (2011). EFSA, Scientific Opinion on Campylobacterin broiler meat production: Control options and performance objectives and/or targets at different stages of the food chain. EFSA J..

[10] Nauta M.J., Johannessen G., Adame L.L., Williams N., Rosenquist H. (2016). The effect of reducing numbers of Campylobacter in broiler intestines on human health risk. Microb. Risk Anal..

[11] Smith S., Messam L.L., Meade J., Gibbons J., McGill K., Bolton D., Whyte P. (2016). The impact of biosecurity and partial depopulation on Campylobacter prevalence in Irish broiler flocks with differing levels of hygiene and economic performance. Infect. Ecol. Epidemiol..

[12] Solis de los Santos F., Donoghue A.M., Venkitanarayanan K., Metcalf J.H., Reyes-Herrera I., Dirain M.L., Aguiar V.F., Blore P.J., Donoghue D.J. (2009). The natural feed additive caprylic acid decreases Campylobacter jejuni colonization in market-aged broiler chickens. Poult. Sci..

[13] Skånseng B., Kaldhusdal M., Moen B., Gjevre A.-G., Johannessen G., Sekelja M., Trosvik P., Rudi K. (2010). Prevention of intestinal Campylobacter jejuni colonization in broilers by combinations of in-feed organic acids. J. Appl. Microbiol..

[14] Hermans D., Martel A., Van Deun K., Verlinden M., Van Immerseel F., Garmyn A., Messens W., Heyndrickx M., Haesebrouck F., Pasmans F. (2010). Intestinal mucus protects Campylobacter jejuni in the ceca of colonized broiler chickens against the bactericidal effects of medium-chain fatty acids. Poult. Sci..

[15] Metcalf J.H., Donoghue A.M., Venkitanarayanan K., Reyes-Herrera I., Aguiar V.F., Blore P.J., Donoghue D.J. (2011). Water administration of the medium-chain fatty acid caprylic acid produced variable efficacy against enteric Campylobacter colonization in broilers1,2. Poult. Sci..

[16] Kittler S., Fischer S., Abdulmawjood A., Glünder G., Klein G. (2013). Effect of Bacteriophage Application on Campylobacter jejuni Loads in Commercial Broiler Flocks. Appl. Environ. Microbiol..

[17] Ghareeb K., Awad W.A., Mohnl M., Porta R., Biarnés M., Böhm J., Schatzmayr G. (2012). Evaluating the efficacy of an avian-specific probiotic to reduce the colonization ofCampylobacter jejuni in broiler chickens. Poult. Sci..

[18] Saint-Cyr M.J., Haddad N., Taminiau B., Poezevara T., Quesne S., Amelot M., Daube G., Chemaly M., Dousset X., Guyard-Nicodème M. (2017). Use of the potential probiotic strain Lactobacillus salivarius SMXD51 to control Campylobacter jejuni in broilers. Int. J. Food Microbiol..

[19] Stern N.J., Eruslanov B.V., Pokhilenko V.D., Kovalev Y.N., Volodina L.L., Perelygin V.V., Mitsevich E.V., Mitsevich I.P., Borzenkov V.N., Levchuk V.P. (2008). Bacteriocins reduce Campylobacter jejuni colonization while bacteria producing bacteriocins are ineffective. Microb. Ecol. Heal. Dis..

[20] Buckley A.M., Wang J., Hudson D.L., Grant A.J., Jones M.A., Maskell D.J., Stevens M.P. (2010). Evaluation of live-attenuated Salmonella vaccines expressing Campylobacter antigens for control of C. jejuni in poultry. Vaccine.

[21] Łaniewski P., Lis M., Wyszyńska A., Majewski P., Godlewska R., Jagusztyn-Krynicka E.K. (2012). Assessment of chicken protection against Campylobacter jejuni infection by immunization with avirulent Salmonella enterica sv. Typhimurium strain producing Campylobacter CjaD/Pal protein. Vaccine Dev. Ther..

[22] Layton S.L., Morgan M.J., Cole K., Kwon Y.M., Donoghue D.J., Hargis B.M., Pumford N.R. (2011). Evaluation of Salmonella-vectored Campylobacter peptide epitopes for reduction of Campylobacter jejuni in broiler chickens. Clin. Vaccine Immunol..

[23] Rickaby B., Eng N.F., Flint A., Stintzi A., Diaz-Mitoma F. (2015). The Application of a Proteoliposome Adjuvant System in the Development of a Campylobacter jejuni Vaccine. Procedia Vaccinol..

[24] Saxena M., John B., Mu M., Van T.T.H., Taki A., Coloe P.J., Smooker P.M. (2013). Strategies to Reduce Campylobacter Colonisation in Chickens. Procedia Vaccinol..

[25] Neal-McKinney J.M., Samuelson D.R., Eucker T.P., Nissen M.S., Crespo R., Konkel M.E. (2014). Reducing Campylobacter jejuni Colonization of Poultry via Vaccination. PLoS ONE.

[26] Wyszyńska A., Raczko A., Lis M., Jagusztyn-Krynicka E.K. (2004). Oral immunization of chickens with avirulent Salmonella vaccine strain carrying C. jejuni 72Dz/92 cjaA gene elicits specific humoral immune response associated with protection against challenge with wild-type Campylobacter. Vaccine.

[27] Clark J.D., Oakes R.D., Redhead K., Crouch C.F., Francis M.J., Tomley F.M., Blake D.P. (2012). Eimeria species parasites as novel vaccine delivery vectors: Anti-Campylobacter jejuni protective immunity induced by Eimeria tenella-delivered CjaA. Vaccine.

[28] Zeng X., Xu F., Lin J. (2010). Development and Evaluation of CmeC Subunit Vaccine against Campylobacter jejuni. J. Vaccines Vaccin..

[29] Rice B.E., Rollins D.M., Mallinson E.T., Carr L., Joseph S.W. (1997). Campylobacter jejuni in broiler chickens: Colonization and humoral immunity following oral vaccination and experimental infection. Vaccine.

[30] Łaniewski P., Kuczkowski M., Chrząstek K., Woźniak A., Wyszyńska A., Wieliczko A., Jagusztyn-Krynicka E.K. (2013). Evaluation of the immunogenicity of Campylobacter jejuni CjaA protein delivered by Salmonella enterica sv. Typhimurium strain with regulated delayed attenuation in chickens. World J. Microbiol. Biotechnol..

[31] Moher D., Shamseer L., Clarke M.G.D., Liberati A., Petticrew M., Shekelle P., Stewart L.A., Group P.-P. (2015). Preferred reporting items for systematic review and meta-analysis protocols (PRISMA-P) 2015 statement. Syst. Rev..

[32] Han Z., Willer T., Pielsticker C., Gerzova L., Rychlik I., Rautenschlein S. (2016). Differences in host breed and diet influence colonization by Campylobacter jejuni and induction of local immune responses in chicken. Gut Pathog..

[33] Humphrey S., Chaloner G., Kemmett K., Davidson N., Williams N., Kipar A., Humphrey T., Wigley P. (2014). Campylobacter jejuni Is Not Merely a Commensal in Commercial Broiler Chickens and Affects Bird Welfare. mBio.

[34] Rosenquist H., Nielsen N.L., Sommer H.M., Nørrung B., Christensen B.B. (2003). Quantitative risk assessment of human campylobacteriosis associated with thermophilic Campylobacter species in chickens. Int. J. Food Microbiol..

[35] De la Cruz M., Conrado I., Nault A., Perez A., Dominguez L., Alvarez J. (2017). Vaccination as a control strategy against Salmonella infection in pigs: A systematic review and meta-analysis of the literature. Res. Veter. Sci..

[36] Osterholm M.T., Kelley N.S., Sommer A., Belongia A.E. (2012). Efficacy and effectiveness of influenza vaccines: A systematic review and meta-analysis. Lancet Infect. Dis..

[37] Lund M., Nordentoft S., Pedersen K., Madsen M. (2004). Detection of Campylobacter spp. in Chicken Fecal Samples by Real-Time PCR. J. Clin. Microbiol..

[38] Weinberg G.A., Szilagyi P.G. (2010). Vaccine Epidemiology: Efficacy, Effectiveness, and the Translational Research Roadmap. J. Infect. Dis..

[39] Hsu S.-M., Chen T.H.-H., Wang C.-H. (2010). Efficacy of Avian Influenza Vaccine in Poultry: A Meta-analysis. Avian Dis..

[40] Bewick V., Cheek L., Ball J. (2004). Statistics review 11: Assessing risk. Crit. Care.

[41] Engels E.A., E Falagas M., Lau J., Bennish M.L. (1998). Typhoid fever vaccines: A meta-analysis of studies on efficacy and toxicity. BMJ.

[42] Basta N.E., Halloran M.E., Matrajt L., Longini I.M. (2008). Estimating Influenza Vaccine Efficacy From Challenge and Community-based Study Data. Am. J. Epidemiol..

[43] R Core Team (2018). R: A Language and Environment for Statistical Computing.

[44] Adams L.J., Zeng X., Lin J. (2019). Development and Evaluation of Two Live Salmonella-Vectored Vaccines for Campylobacter Control in Broiler Chickens. Foodborne Pathog. Dis..

[45] Liu X., Adams L.J., Zeng X., Lin J. (2019). Evaluation of in ovo vaccination of DNA vaccines for Campylobacter control in broiler chickens. Vaccine.

[46] Nothaft H., Perez-Munoz M.E., Gouveia G.J., Duar R.M., Wanford J.J., Lango-Scholey L., Panagos C.G., Srithayakumar V., Plastow G.S., Coros C. (2017). Co-administration of the Campylobacter jejuni N-glycan based vaccine with probiotics improves vaccine performance in broiler chickens. Appl. Environ. Microbiol..

[47] Annamalai T., Pina-Mimbela R., Kumar A., Binjawadagi B., Liu Z., Renukaradhya G.J., Rajashekara G. (2013). Evaluation of nanoparticle-encapsulated outer membrane proteins for the control of Campylobacter jejuni colonization in chickens. Poult. Sci..

[48] Gorain C., Singh A., Bhattacharyya S., Kundu A., Lahiri A., Gupta S., Mallick A.I. (2020). Mucosal delivery of live Lactococcus lactis expressing functionally active JlpA antigen induces potent local immune response and prevent enteric colonization of Campylobacter jejuni in chickens. Vaccine.

[49] Hodgins D.C., Barjesteh N., Paul M.S., Ma Z., A Monteiro M., Sharif S. (2015). Evaluation of a polysaccharide conjugate vaccine to reduce colonization by Campylobacter jejuni in broiler chickens. BMC Res. Notes.

[50] Meunier M., Guyard-Nicodème M., Vigouroux E., Poezevara T., Beven V., Quesne S., Bigault L., Amelot M., Dory D., Chemaly M. (2017). Promising new vaccine candidates against Campylobacter in broilers. PLoS ONE.

[51] Meunier M., Guyard-Nicodème M., Vigouroux E., Poezevara T., Béven V., Quesne S., Amelot M., Parra A., Chemaly M., Dory D. (2018). A DNA prime/protein boost vaccine protocol developed against Campylobacter jejuni for poultry. Vaccine.

[52] Okamura M., Tominaga A., Ueda M., Ohshima R., Kobayashi M., Tsukada M., Yokoyama E., Takehara K., Deguchi K., Honda T. (2012). Irrelevance between the Induction of Anti-Campylobacter Humoral Response by a Bacterin and the Lack of Protection against Homologous Challenge in Japanese Jidori Chickens. J. Veter Med. Sci..

[53] Radomska K.A., Vaezirad M.M., Verstappen K.M., Wösten M.M.S.M., Wagenaar J.A., Van Putten J.P.M. (2016). Chicken Immune Response after In Ovo Immunization with Chimeric TLR5 Activating Flagellin of Campylobacter jejuni. PLoS ONE.

[54] Singh A., Nisaa K., Bhattacharyya S., Mallick A.I. (2019). Immunogenicity and protective efficacy of mucosal delivery of recombinant hcp of Campylobacter jejuni Type VI secretion system (T6SS) in chickens. Mol. Immunol..

[55] Taha-Abdelaziz K., Hodgins D.C., Alkie T.N., Quinteiro-Filho W., Yitbarek A., Astill J., Sharif S. (2018). Oral administration of PLGA-encapsulated CpG ODN and Campylobacter jejuni lysate reduces cecal colonization by Campylobacter jejuni in chickens. Vaccine.

[56] Theoret J.R., Cooper K.K., Zekarias B., Roland K.L., Law B.F., Curtiss R., Joens L.A. (2012). The Campylobacter jejuni Dps homologue is important for in vitro biofilm formation and cecal colonization of poultry and may serve as a protective antigen for vaccination. Clin. Vaccine Immunol..

[57] Wang C., Zhou H., Guo F., Yang B., Su X., Lin J., Xu F. (2020). Oral Immunization of Chickens with Lactococcus lactis Expressing cjaA Temporarily Reduces Campylobacter jejuni Colonization. Foodborne Pathog. Dis..

[58] Yang Y., Wolfenden A., Mandal R.K., Faulkner O., Hargis B., Kwon Y.M., Bielke L. (2017). Evaluation of recombinant Salmonella vaccines to provide cross-serovar and cross-serogroup protection. Poult. Sci..

[59] Kakuda T., DiRita V.J. (2006). Cj1496c Encodes a Campylobacter jejuni Glycoprotein That Influences Invasion of Human Epithelial Cells and Colonization of the Chick Gastrointestinal Tract. Infect. Immun..

[60] Konkel M.E., Gray S.A., Kim B.J., Garvis S.G., Yoon J. (1999). Identification of the EnteropathogensCampylobacter jejuni and Campylobacter coli Based on the cadF Virulence Gene and Its Product. J. Clin. Microbiol..

[61] Konkel M.E., Kim B.J., Rivera-Amill V., Garvis S.G. (1999). Bacterial secreted proteins are required for the internalization of Campylobacter jejuni into cultured mammalian cells. Mol. Microbiol..

[62] Jin S., Joe A., Lynett J., Hani E.K., Sherman P.C., Chan V.L. (2001). JlpA, a novel surface-exposed lipoprotein specific to Campylobacter jejuni, mediates adherence to host epithelial cells. Mol. Microbiol..

[63] Keo T., Collins J., Kunwar P., Blaser M.J., Iovine N.M. (2011). Campylobacter capsule and lipooligosaccharide confer resistance to serum and cationic antimicrobials. Virulence.

[64] Lin J., Michel L.O., Zhang Q. (2002). CmeABC Functions as a Multidrug Efflux System in Campylobacter jejuni. Antimicrob. Agents Chemother..

[65] Müller A., Thomas G.H., Horler R., Brannigan J.A., Blagova E., Levdikov V.M., Fogg M.J., Wilson K.S., Wilkinson A.J. (2005). An ATP-binding cassette-type cysteine transporter in Campylobacter jejuni inferred from the structure of an extracytoplasmic solute receptor protein. Mol. Microbiol..

[66] Konkel M.E., Larson C.L., Flanagan R.C. (2009). Campylobacter jejuni FlpA Binds Fibronectin and Is Required for Maximal Host Cell Adherence. J. Bacteriol..

[67] Fernando U., Biswas D., Allan B., Willson P., Potter A.A. (2007). Influence of Campylobacter jejuni fliA, rpoN and flgK genes on colonization of the chicken gut. Int. J. Food Microbiol..

[68] Neal-McKinney J.M., Konkel M.E. (2012). The Campylobacter jejuni CiaC virulence protein is secreted from the flagellum and delivered to the cytosol of host cells. Front. Cell. Infect. Microbiol..

[69] Nachamkin I., Yang X.H., Stern N.J. (1993). Role of Campylobacter jejuni flagella as colonization factors for three-day-old chicks: Analysis with flagellar mutants. Appl. Environ. Microbiol..

[70] Wassenaar T.M., Van Der Zeijst B.A.M., Ayling R., Newell D.G. (1993). Colonization of chicks by motility mutants of Campylobacter jejuni demonstrates the importance of flagellin A expression. J. Gen. Microbiol..

[71] Liaw J., Hong G., Davies C., Elmi A., Sima F., Stratakos A., Stef L., Pet I., Hachani A., Corcionivoschi N. (2019). The Campylobacter jejuni Type VI Secretion System Enhances the Oxidative Stress Response and Host Colonization. Front. Microbiol..

[72] Lertpiriyapong K., Gamazon E.R., Feng Y., Park D.S., Pang J., Botka G., Graffam M.E., Ge Z., Fox J.G. (2012). Campylobacter jejuni Type VI Secretion System: Roles in Adaptation to Deoxycholic Acid, Host Cell Adherence, Invasion, and In Vivo Colonization. PLoS ONE.

[73] Alemka A., Nothaft H., Zheng J., Szymanski C.M. (2013). N-Glycosylation of Campylobacter jejuni Surface Proteins Promotes Bacterial Fitness. Infect. Immun..

[74] Karlyshev A.V., Everest P., Linton D., A Cawthraw S., Newell D.G., Wren B.W. (2004). The Campylobacter jejuni general glycosylation system is important for attachment to human epithelial cells and in the colonization of chicks. Microbiology.

[75] Chart H., Frost J.A., Conway D., Rowe B. (1996). Outer membrane characteristics of Campylobacter jejuni grown in chickens. FEMS Microbiol. Lett..

[76] Godlewska R., Wisniewska K., Pietras Z., Jagusztyn-Krynicka E.K. (2009). Peptidoglycan-associated lipoprotein (Pal) of Gram-negative bacteria: Function, structure, role in pathogenesis and potential application in immunoprophylaxis. FEMS Microbiol. Lett..

[77] Konkel M.E., A Joens L. (1989). Adhesion to and invasion of HEp-2 cells by Campylobacter spp.. Infect. Immun..

[78] Khoury C.A., Meinersmann R.J. (1995). A Genetic Hybrid of the Campylobacter jejuni flaA Gene with LT-B of Escherichia coli and Assessment of the Efficacy of the Hybrid Protein as an Oral Chicken Vaccine. Avian Dis..

[79] Godlewska R., Kuczkowski M., Wyszyńska A., Klim J., Derlatka K., Woźniak-Biel A., Jagusztyn-Krynicka E.K. (2016). Evaluation of a protective effect of in ovo delivered Campylobacter jejuni OMVs. Appl. Microbiol. Biotechnol..

[80] Kobierecka P.A., Wyszyńska A.K., Gubernator J., Kuczkowski M., Wiśniewski O., Maruszewska M., Wojtania A., Derlatka K.E., Adamska I., Godlewska R. (2016). Chicken Anti-Campylobacter Vaccine–Comparison of Various Carriers and Routes of Immunization. Front. Microbiol..

[81] Chintoan-Uta C., Cassady-Cain R.L., Al-Haideri H., Watson E., Kelly D.J., Smith D.G., Sparks N.H., Kaiser P., Stevens M.P. (2015). Superoxide dismutase SodB is a protective antigen against Campylobacter jejuni colonisation in chickens. Vaccine.

[82] Kobierecka P.A., Olech B., Ksiazek M., Derlatka K., Adamska I., Majewski P.M., Jagusztyn-Krynicka E.K., Wyszynska A.K. (2016). Cell Wall Anchoring of the Campylobacter Antigens to Lactococcus lactis. Front. Microbiol..

[83] Bennett C.E., Thomas R., Williams M., Zalasiewicz J., Edgeworth M., Miller H., Coles B., Foster A., Burton E.J., Marume U. (2018). The broiler chicken as a signal of a human reconfigured biosphere. R. Soc. Open Sci..

[84] Souillard R., Répérant J.-M., Experton C., Huneau-Salaun A., Coton J., Balaine L., Le Bouquin S. (2019). Husbandry Practices, Health, and Welfare Status of Organic Broilers in France. Animals.

[85] Lacharme-Lora L., Chaloner G., Gilroy R., Humphrey S., Gibbs K., Jopson S., Wright E., Reid W., Ketley J., Humphrey T. (2017). B lymphocytes play a limited role in clearance of Campylobacter jejuni from the chicken intestinal tract. Sci. Rep..

[86] El-Shibiny A., Connerton P.L., Connerton I.F. (2005). Enumeration and Diversity of Campylobacters and Bacteriophages Isolated during the Rearing Cycles of Free-Range and Organic Chickens. Appl. Environ. Microbiol..

[87] Pumtang-On P., Mahony T.J., Hill R.A., Pavic A., Vanniasinkam T. (2020). Investigation of Campylobacter colonization in three Australian commercial free-range broiler farms. Poult. Sci..

[88] Sahin O., Luo N., Huang S., Zhang Q. (2003). Effect of Campylobacter-Specific Maternal Antibodies on Campylobacter jejuni Colonization in Young Chickens. Appl. Environ. Microbiol..

[89] Wesley R.D., Lager K.M. (2006). Overcoming maternal antibody interference by vaccination with human adenovirus 5 recombinant viruses expressing the hemagglutinin and the nucleoprotein of swine influenza virus. Veter Microbiol..

[90] Zhang F., Peng B., Chang H., Zhang R., Lu F., Wang F., Fang F., Chen Z. (2016). Intranasal Immunization of Mice to Avoid Interference of Maternal Antibody against H5N1 Infection. PLoS ONE.

[91] Bublot M., Pritchard N., Le Gros F.-X., Goutebroze S. (2007). Use of a Vectored Vaccine against Infectious Bursal Disease of Chickens in the Face of High-Titred Maternally Derived Antibody. J. Comp. Pathol..

